# Habitability on Early Mars and the Search for Biosignatures with the ExoMars Rover

**DOI:** 10.1089/ast.2016.1533

**Published:** 2017-07-01

**Authors:** Jorge L. Vago, Frances Westall, Andrew J. Coates, Ralf Jaumann, Oleg Korablev, Valérie Ciarletti, Igor Mitrofanov, Jean-Luc Josset, Maria Cristina De Sanctis, Jean-Pierre Bibring, Fernando Rull, Fred Goesmann, Harald Steininger, Walter Goetz, William Brinckerhoff, Cyril Szopa, François Raulin, Frances Westall, Howell G. M. Edwards, Lyle G. Whyte, Alberto G. Fairén, Jean-Pierre Bibring, John Bridges, Ernst Hauber, Gian Gabriele Ori, Stephanie Werner, Damien Loizeau, Ruslan O. Kuzmin, Rebecca M. E. Williams, Jessica Flahaut, François Forget, Jorge L. Vago, Daniel Rodionov, Oleg Korablev, Håkan Svedhem, Elliot Sefton-Nash, Gerhard Kminek, Leila Lorenzoni, Luc Joudrier, Viktor Mikhailov, Alexander Zashchirinskiy, Sergei Alexashkin, Fabio Calantropio, Andrea Merlo, Pantelis Poulakis, Olivier Witasse, Olivier Bayle, Silvia Bayón, Uwe Meierhenrich, John Carter, Juan Manuel García-Ruiz, Pietro Baglioni, Albert Haldemann, Andrew J. Ball, André Debus, Robert Lindner, Frédéric Haessig, David Monteiro, Roland Trautner, Christoph Voland, Pierre Rebeyre, Duncan Goulty, Frédéric Didot, Stephen Durrant, Eric Zekri, Detlef Koschny, Andrea Toni, Gianfranco Visentin, Martin Zwick, Michel van Winnendael, Martín Azkarate, Christophe Carreau

**Affiliations:** ^1^ ESA/ESTEC, Noordwijk, the Netherlands.; ^2^ CNRS-OSUC-Centre de Biophysique Moléculaire, Orléans, France.; ^3^ Mullard Space Science Laboratory (MSSL), University College London, United Kingdom.; ^4^ DLR Institut für Planetenforschung, Berlin, Germany.; ^5^ Space Research Institute of the Russian Academy of Sciences (IKI), Moscow, Russia.; ^6^ LATMOS/IPSL, UVSQ Université Paris-Saclay, UPMC Université Paris 06, CNRS, Guyancourt, France.; ^7^ SPACE-X, Space Exploration Institute, Neuchâtel, Switzerland.; ^8^ Istituto di Astrofisica e Planetologia Spaziali INAF, Roma, Italy.; ^9^ Institut d'Astrophysique Spatiale (IAS), Orsay, France.; ^10^ Unidad Asociada UVA-CSIC, Universidad de Valladolid, Spain.; ^11^ Max-Planck-Institut für Sonnensystemforschung (MPS), Göttingen, Germany.; ^12^ NASA Goddard Space Flight Center, Greenbelt MD, United States.; ^13^ Université Paris-Est Créteil, Laboratoire Interuniversitaire des Systèmes Atmosphériques (LISA), Paris, France.; ^14^ University of Bradford, United Kingdom.; ^15^ McGill University, Ste. Anne de Bellevue, Quebec, Canada.; ^16^ Centro de Astrobiología (CAB), Madrid, Spain.; ^17^ Space Research Centre, University of Leicester, United Kingdom.; ^18^ DLR Institut für Planetenforschung, Berlin, Germany.; ^19^ International Research School of Planetary Physics (IRSPS), Pescara, Italy.; ^20^ Centre for Earth Evolution and Dynamics, University of Oslo, Norway.; ^21^ Université Lyon 1, Ens de Lyon, CNRS, Villeurbanne, France.; ^22^ Vernadsky Institute, Russian Academy of Sciences, Moscow, Russia.; ^23^ Planetary Science Institute, Waunakee WI, United States.; ^24^ Institut de Recherche en Astrophysique et Planétologie (IRAP), Toulouse, France.; ^25^ Laboratoire de Météorologie Dynamique (LMD), Institut Pierre Simon Laplace Université Paris 6, Paris, France.; ^26^ TsNIIMash, Korolev, Russia.; ^27^ NPO S. Lavochkin, Khimki, Russia.; ^28^ Thales Alenia Space, Torino, Italy.; ^29^ Université Nice Sophia Antipolis, Institut de Chimie de Nice, Nice, France.; ^30^ CSIC-Universidad de Granada, Spain.; ^31^ Centre National d'Études Spatiales (CNES), Toulouse, France.

## Abstract

The second ExoMars mission will be launched in 2020 to target an ancient location interpreted to have strong potential for past habitability and for preserving physical and chemical biosignatures (as well as abiotic/prebiotic organics). The mission will deliver a lander with instruments for atmospheric and geophysical investigations and a rover tasked with searching for signs of extinct life. The ExoMars rover will be equipped with a drill to collect material from outcrops and at depth down to 2 m. This subsurface sampling capability will provide the best chance yet to gain access to chemical biosignatures. Using the powerful Pasteur payload instruments, the ExoMars science team will conduct a holistic search for traces of life and seek corroborating geological context information. Key Words: Biosignatures—ExoMars—Landing sites—Mars rover—Search for life. Astrobiology 17, 471–510.

**Table of Contents**
1. Article Organization
2. Introduction
  2.1. ExoMars origin
  2.2. A difficult adolescence
  2.3. Joint program
3. Early Mars as an Exobiology Target
  3.1. A first window of opportunity for life
  3.2. Separate ways
    3.2.1. Young Earth
    3.2.2. Young Mars
    3.2.3. Young Venus
  3.3. Lessons for ExoMars: when and where?
4. Biosignatures: Which and How Reliable?
  4.1. Morphological biosignatures
  4.2. Chemical biosignatures
    4.2.1. Isomerism selectivity
    4.2.2. Molecular weight fingerprints
    4.2.3. Bulk isotopic fractionation
  4.3. Importance of geological context for boosting biosignature confidence
  4.4. Life's decision points
  4.5. Examples using the ExoMars biosignature score
    4.5.1. Kitty's Gap, N.W. Australia
    4.5.2. Josefsdal Chert, Barberton, South Africa
    4.5.3. Martian Meteorite ALH84001
    4.5.4. Yellowknife Bay, Mars
5. The Martian Environment and the Need for Subsurface Exploration
  5.1. Results from previous missions
  5.2. Degradation of organic matter
  5.3. Access to molecular biosignatures
6. The ExoMars Rover and Its Pasteur Payload
  6.1. From panoramic to molecular scale through nested investigations
  6.2. Pasteur payload instruments
    6.2.1. Panoramic camera system
    6.2.2. IR spectrometer
    6.2.3. Shallow ground-penetrating radar
    6.2.4. Subsurface neutron detector
    6.2.5. Close-up imager
    6.2.6. Drill IR spectrometer
    6.2.7. Subsurface drill
    6.2.8. Sample preparation and distribution system
    6.2.9. MicrOmega
    6.2.10. Raman laser spectrometer
    6.2.11. Mars organic molecule analyzer
  6.3. The reference surface mission
7. A Suitable Landing Site
  7.1. Scientific constraints
  7.2. Engineering constraints
  7.3. Planetary protection constraints
  7.4. Possible locations for landing
    7.4.1. Oxia Planum (18.159°N, 335.666°E; −3 km MOLA)
    7.4.2. Mawrth Vallis (22.160°N, 342.050°E; −2 km MOLA)
8. Conclusions
Acknowledgments
Author Disclosure Statement
References
Abbreviations Used

## 1. Article Organization

This is the introduction article in a collection dedicated to the ExoMars rover. Starting from a discussion of the mission's science underpinnings, we describe the rover and its Pasteur payload, drill and sample processing system, and present the reference surface exploration scenario. We conclude by addressing the desirable scientific attributes of the landing site region and the limits on various terrain properties imposed by engineering constraints. Dedicated articles about each of the instruments can also be found in this issue.

## 2. Introduction

Discovering life elsewhere is one of the great scientific challenges of our time. We can begin to address this by exploring Mars, an object that shared with Earth a similar early geological history, particularly during the time when life is supposed to have appeared on our planet.

### 2.1. ExoMars origin

The beginnings of the ExoMars rover can be traced to 1996, when ESA tasked an exobiology science team with formulating guidelines for future search-for-life missions in the Solar System. This group was active during 1997–1998, an exciting period in Mars exploration; following a 20-year hiatus after the Viking missions, Pathfinder had landed with an interesting new element: a rover. The team published their findings in what is now known as the “Red Book Report” (Brack *et al.*, 1999; Westall *et al.*, 2000). A major outcome was the recommendation to seek evidence of extinct life below the surface of Mars.

The team identified three fundamental requirements: (1) that the landing area possess high exobiology interest—ancient sites containing aqueous sedimentary or hydrothermal deposits relatively free from dust would constitute prime targets; (2) that samples free from surface oxidation and radiation damage be collected at several locations by a rover equipped with a drill capable of reaching well below the soil and into surface rocks; and (3) that an integral set of measurements be performed at each site, and on each sample to achieve a comprehensive understanding of petrology, mineralogy, and geochemistry (geological context) and thus inform the search for biosignatures.

After the release of the Red Book Report, ESA undertook a series of feasibility studies for mission concepts and integrated payload systems.

### 2.2. A difficult adolescence

In 2001, exobiology at ESA received a boost when European ministers approved the Aurora Program with the goal to devise and implement a plan to explore Solar System bodies holding promise for life (Horneck *et al.*, 2016). ESA assessed a range of options in cooperation with the scientific community. Two Mars missions were identified as necessary before any future human endeavor: the ExoMars rover and Mars sample return (MSR), the latter most likely as part of an international effort. During 2002, at its concurrent design facility (CDF), ESA completed a preliminary architecture study for ExoMars. In 2003, the agency released a call for instruments for the rover's Pasteur payload. Phase A studies followed in 2004. The ExoMars mission (Baglioni *et al.*, 2006; Vago *et al.*, 2006; Vago and Kminek, 2008) was approved at the 2005 ESA Ministerial Conference. However, a last-minute request to accommodate an instrumented station on the landing platform—to recover science from the discontinued Netlander mission (Dehant *et al.*, 2004)—resulted in a more complicated design, requiring a larger launcher, which could not be achieved with the available budget. Nevertheless, the project team was instructed to begin the technical work; the rest of the funding would be provided at the 2008 ESA Ministerial Conference. A number of studies were necessary to redefine the new mission's more ambitious scope, and thus the target 2009 launch date was postponed, first to 2011, then to 2013.

Unfortunately, the anticipated additional financial injection did not materialize because of the nascent economic crisis. At the same time, NASA was experiencing difficulties with the Mars Science Laboratory (MSL) project, which affected their ability to prepare a new mission for 2016. In 2009, ESA and NASA agreed that they could accomplish more by uniting forces. A scenario was outlined for a joint program that would have as ultimate goal an international MSR mission in the mid to late 2020s. Within this program, the agencies defined the first two missions for launch in 2016 and 2018. Regretfully, budget constraints in the United States resulted in NASA having to scale down its participation. To help resolve this situation, ESA, NASA, and Roscosmos met in late 2011 to discuss implementing the joint program as a tripartite collaboration, but shortly thereafter NASA informed ESA and Roscosmos that they would no longer be able to contribute major mission elements. After a program reassessment phase, ESA and Roscosmos signed a cooperation agreement in 2013 to work in partnership to develop and launch the two ExoMars missions.

### 2.3. Joint program

The first ExoMars mission was launched on March 14, 2016, from the Baikonur cosmodrome, in Kazakhstan, and arrived at Mars on October 19, 2016. It consists of two major elements: the Trace Gas Orbiter (TGO) and the Schiaparelli entry, descent, and landing demonstrator module (EDM). The objective of TGO is to conduct a detailed analysis of atmospheric gases, including methane (CH_4_) and other minor constituents (Allen *et al.*, 2006; Sherwood Lollar *et al.*, 2006; Yung *et al.*, 2010; Yung and Chen, 2015), and study the surface to seek signatures of possible active processes; TGO will also serve as a communications relay for surface missions until the end of 2022. The EDM's goal was to prove technologies for controlled landing and perform surface measurements. Unfortunately, the last phase of the landing sequence did not work and the lander was lost.

The second mission will deliver a rover tasked with searching for signs of past life; however, its payload also has the potential to recognize chemical indicators of extant life. The ExoMars rover will drill to depths of 2 m to collect and analyze samples that have been shielded from the harsh conditions that prevail at the surface, where radiation and oxidants can destroy organic compounds. The lander will be equipped with instruments devoted to atmospheric and geophysical investigations.

ESA and Roscosmos agreed a balanced sharing of responsibilities for the different elements. ESA would provide the TGO and EDM for the first mission, and the carrier and rover for the second. Roscosmos would furnish both launchers and be in charge of the second mission's descent module. NASA would also deliver important contributions to ExoMars, such as the Electra ultra high frequency (UHF) radio package for TGO-to-Mars-surface proximity link communications, engineering support to the EDM, and a major part of Mars organic molecule analyzer (MOMA), the organic molecule characterization instrument on the rover.

## 3. Early Mars as an Exobiology Target

If life ever arose on the red planet, it probably did when Mars was wetter, sometime within the first half billion years after planetary formation (Nisbet and Sleep, 2001; Zahnle *et al.*, 2007). Conditions then were similar to those when microbes gained a foothold on the young Earth. This marks Mars as a primary target to search for signs of life in our Solar System. The knowledge we have gathered about early Earth environments and biosignatures has been extremely useful (Fairén *et al.*, 2010; Westall, 2012; Westall *et al.*, 2013). We briefly discuss the rocky planets to better frame how their evolution may have affected the availability of liquid water; the timing of opportunities for prebiotic chemistry; and the possible emergence of life, its distribution, and its preservation record accessibility in the context of a Mars rover mission.

### 3.1. A first window of opportunity for life

Although Earth, Venus, and Mars formed mainly from locally sourced material, the final stages of accretion blurred chemical differences by integrating contributions from elsewhere—Jupiter and Saturn's wanderings scattered objects in the region presently occupied by the asteroid belt and beyond, delivering water and other volatiles (including prebiotic chemicals) not found in planetesimals formed closer to the protostar (Morbidelli *et al.*, 2000; Albarède, 2009; Alexander *et al.*, 2012; Marty *et al.*, 2013; DeMeo and Carry, 2014; Hallis *et al.*, 2015; Grazier, 2016; Meinert *et al.*, 2016).

**FIG. 1.**
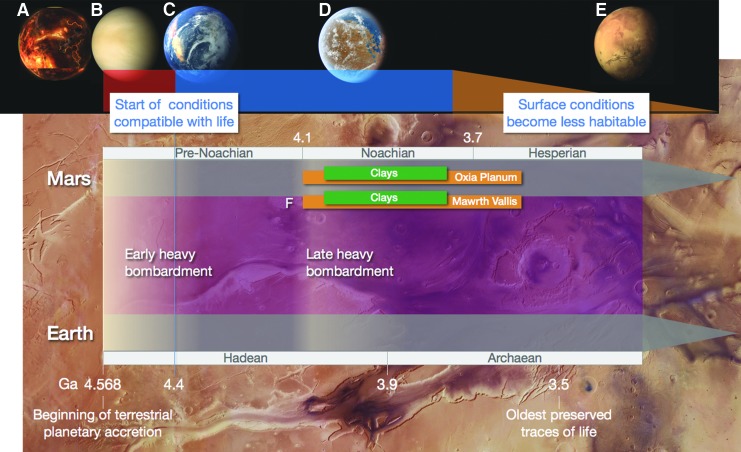
Sketch of terrestrial planet evolution applied to early Mars and Earth. **(A)** Very high temperatures developed during accretion. **(B)** As they cooled down, rocky planets outgassed supercritical atmospheres. **(C)** Global oceans formed once atmospheric water could condense. **(D)** Each planet followed a separate path; Mars maintained some surface liquid water through most of the Noachian. A possible window of opportunity for life opened once water temperatures dropped <80°C (indicated by the blue bar on top). We show with a tapering orange bar the onset of (gradual) change toward less habitable conditions. **(E)** Modern Mars is a very cold, desert-like planet. Subtle white shading represents the relative intensity of meteoritic delivery to the inner Solar System. To maximize our chances of finding signs of past life, we must target the “sweet spot” in Mars' geological history—the early Noachian. **(F)** The approximate age of the deposits (orange bars) and main targets of interest (superimposed green bars) at the two ExoMars candidate landing sites.

Several tens of million years in the making (Fig. 1A), the three terrestrial planets were giant magma spheres that included traces of water retained through their formation process (Elkins-Tanton, 2013). Differentiation kicked in early on (Boyet and Carlson, 2005); dense constituents, radioactive and otherwise, sunk, giving rise to the cores, whereas the lighter silicates and volatiles surged to form the mantles (Elkins-Tanton, 2012). Initially, surface temperatures were a torrid 1800–2000 K. Molten landscapes oozed slowly, with bits of scum floating here and there. The heat flow coming from the interior was high, in the order of 140 W/m^2^ (Sleep, 2010). In the case of Earth, massive tidal heating from the nearby Moon compounded this effect (Zahnle *et al.*, 2007; Sleep *et al.*, 2014).

As the planets cooled, their mantles degassed extensively (Elkins-Tanton, 2008; Hirschmann and Kohlstedt, 2012) and dense, several-hundred-bar (mainly water (H_2_O), carbon dioxide (CO_2_), and nitrogen), supercritical atmospheres developed very quickly (Zahnle *et al.*, 2010) (Fig. 1B). In another 20 Myr or so, the internal heat flow would have waned to about 0.5 W/m^2^ (Sleep, 2010). By then mantles and crusts would have become solid. On each of the three planets, the temperature of the very hot, thick atmosphere would eventually drop below the critical point (Elkins-Tanton, 2011)—for pure water, 647 K and 221 bar. Phase change processes occur suddenly. At a moment's notice, a hot ocean, many hundreds of meters deep, rained from the sky. Because Mars is smaller, and hence cooled faster, it is reasonable to assume that the phase-differentiation deluge happened there first. Venus and Earth, in contrast, lost their heat more slowly. Since our sister planet was closer to the Sun, we will posit that a hot ocean developed next on Earth and shortly thereafter on Venus. The residual atmospheres were still dense pressure cookers, supporting ocean temperatures of a few hundred degrees centigrade.

Nobody knows how long these oceans persisted, but as their temperature became more clement, they would open a first window of opportunity for prebiotic chemistry on Mars (first), Earth, and Venus (Fig. 1C, D).

### 3.2. Separate ways

For life to have a chance, our planets had to get rid of their hot gas envelopes while somehow holding on to some surface water. Early atmospheric evolution was complex and involved a number of interacting processes. Sources included mantle outgassing, volcanism, and impact delivery. Among the sinks we have thermal and wave-driven escape, ultraviolet (UV) erosion, solar wind forcing, impact erosion, and mineral sequestration. The timing and relative importance of the various effects are not understood well enough to provide an accurate picture. They depended on planet mass, interior dynamics, atmospheric composition, and distance to the Sun. Investigators have tried to piece together the information available from space missions so far. Attempts to reconcile atmospheric isotopic ratio data with observed mineralogical composition have proven difficult. For very complete discussions, the reader is referred to the works of Zahnle *et al.* (2007), Lammer *et al.* (2008, 2013), and Albarède (2009).

The young Sun's UV heating and photo dissociation at high altitudes split water and ammonia molecules, allowing the lighter H^+^ to escape. Also, despite the widespread belief that having a magnetic field protects a planet against atmospheric loss, the opposite is actually true. On Earth, in the topside auroral ionosphere, H^+^ and the much heavier O^+^ (that normally would be gravitationally bound) regularly escape along geomagnetic field lines through transverse ion acceleration by electrostatic plasma waves and subsequent magnetic focusing into upward traveling ion conics (initially) and beams (later, once the particle velocity distribution has folded more) (Vago *et al.*, 1992; André and Yau, 1997). Sounding rockets are required to study the particle energizing mechanism, which typically takes place at low altitudes of a few hundred kilometers. Satellites moving in higher orbits with speeds of several kilometers per second can sample the ion beams. All planets with a magnetic field and an atmosphere are susceptible to this escape mechanism. The same process was active on early Earth and (probably) Mars (while it had a magnetic field). In fact, the remnant crustal magnetic field on Mars may still be strong enough for upward traveling ion conics to exist. In this case, the Mars Atmosphere and Volatile Evolution Mission (MAVEN) should be able to detect their higher altitude expression: the ion beams. We do not know whether Venus ever had a magnetic field. Perhaps core convection was too weak for its creation (Stevenson, 2003, 2010), but notwithstanding this, our sister planet is able to retain a dense atmospheric envelope. The main reason for Mars' present thin atmosphere is not its lack of magnetic field but its feeble gravity. Despite all these effects, at least in the beginning, the dominant atmospheric escape mechanism for the three planets pointed downward, into the mantle.

Roughly 120 Myr into our Solar System's history—about 4.45 Ga ago—the terrestrial planets were immense engines fueled by their own internal heat. Initial size and rotational rate had primed their inner clock workings. Deep, viscous, convective flow patterns were set in motion that would ultimately shape the evolution of the surface environment. The global oceans were still hot (about 500 K), but their temperature would start to wane (Sleep *et al.*, 2001); this would have occurred much faster in the case of Mars, which was smaller and further away from a faint, young Sun whose luminosity was roughly 70% that of today. Calcium and magnesium carbonate could form in equilibrium with basaltic rocks in the uppermost region of the oceanic crust. For Earth, Sleep (2010) estimated that the available mass of CaO and MgO would have been able to react with up to 10 bar of CO_2_ to form carbonates. To remove any more CO_2_, the planets had to possess the means to dispose of the carbonates and present new crust for reaction with seawater. Several physical processes promoted subseafloor basaltic fracturing, faulting, and permeability (Bercovici and Ricard, 2014). In these early days, tectonic recycling rates were high, submarine volcanism was widespread and very active, not unlike present terrestrial midocean ridge axes chemistry, but distributed over much larger areas (Sleep, 2007). Rapid material turnover, coupled with vigorous hydrothermal circulation, provided the means to sequester much CO_2_ (Sleep and Zahnle, 2001; Tomkinson *et al.*, 2013).

We next consider separately the possibility for life to have arisen *de novo* on Earth, Mars, and Venus, although material including viable life forms could have been transported between planets (Gollihar *et al.*, 2014).

#### 3.2.1. Young Earth

As Earth cooled down, the continuous formation of carbonates on the oceanic crust and their subsequent sagduction (and later, subduction) fixed most of the atmospheric CO_2_ in the planet's deep interior. In contrast, the decomposition of carbonate rocks in the mantle released modest amounts of CO_2_ into the ocean and atmosphere through volcanoes and hotspots, closing a cycle that is still active today (Walker *et al.*, 1981). Much of the greenhouse effect provided by CO_2_ would have disappeared in several tens to a couple of hundred million years (Zahnle *et al.*, 2007; Sleep, 2010; Sleep *et al.*, 2014). Considering the weak Sun illumination, in the absence of some other greenhouse gas, Earth's surface temperature would have quickly plunged to subfreezing values: ∼250 K—see Figure 1A (bottom dashed curve) in the work of Kasting and Ackerman (1986). The period during which the ocean's surface would have remained at clement conditions (10–70°C) would have been necessarily short, of the order of several million years, because maintaining such surface temperatures would have required 3–25 bar of CO_2_, as implied by Figure 1A in the work of Sleep *et al.* (2001).

Although extensive volcanism and the occasional meteoritic impact (Bada et al., 1994) provided numerous localized balmy environments, this extremely cold Earth scenario is in contradiction with ocean temperature values (30–70°C) derived from isotopic measurements performed on the most ancient (3.4–3.8 Ga old) sedimentary rocks preserved (Kasting and Ono, 2006; Hren *et al.*, 2009; Westall, 2012) and on much older (4.0–4.4 Ga old) detrital zircons (Wilde *et al.*, 2001; Valley *et al.*, 2002; Cottin *et al.*, 2015). It is, therefore, likely that an additional atmospheric constituent prevented our roughly 200-Myr-old planet from becoming a frigid snowball (Pavlov *et al.*, 2000; Emmanuel and Ague, 2007; Kasting, 2013). In this context we consider methane. A gradual buildup of CH_4_ could have played a major greenhouse role—perhaps assisted by a low planetary albedo—in countering the effects of a rapidly thinning CO_2_ presence [see Fig. 4 in the work Kasting and Ono (2006) and also Rosing *et al.* (2010)], but then CH_4_ had to be replenished more or less continuously since it is rapidly destroyed by UV photolysis. Although possible, it is improbable that methanogenic archaea could have been responsible; their widespread existence would have implied a large degree of life diversification already 4.4 Ga ago. A more plausible mechanism for the production of copious CH_4_ in a hyperactive young Earth is by abiotic means (Sherwood Lollar *et al.*, 2006).

Geochemical/hydrothermal CH_4_ had to be abundant on early Earth since it was a byproduct of the same successful recipe responsible for the sequestration of atmospheric CO_2_ (Nisbet, 2000; Nisbet and Sleep, 2001; Schulte *et al.*, 2006). As seawater diffused downward through fractured ocean crust, it reacted with mantle host rocks at high temperatures and transformed into a hydrothermal fluid that became enriched in a variety of compounds and depleted in others, depending on the subsurface reaction conditions and the nature of the leached rocks (Konn *et al.*, 2015). The result was emitted in the form of thick, smoke-like underwater plumes distributed ubiquitously.

Among all the possible abiotic mechanisms for CH_4_ generation (Etiope and Sherwood Lollar, 2013; Holm *et al.*, 2015), serpentinization was the most important; that is, the low-temperature (150–400°C) hydrolysis and transformation of ultramafic rocks—ferromagnesian olivine- and pyroxene-group minerals or the Hadean Mg-rich basalts and komatiites (Russell *et al.*, 2014; Shibuya *et al.*, 2015; Sobolev *et al.*, 2016)—which produces H_2_ that can then react with simple oxidized carbon compounds, such as CO_2_ and CO, under reducing conditions to release CH_4_ and other organic molecules through Fischer–Tropsch-type synthesis. Not only did widespread serpentinization play a fundamental greenhouse role on early Earth, it also contributed the bricks and mortar for many prebiotic reactions that, in time, could have led to the first proto-organisms (Russell and Hall, 1997; Kelley *et al.*, 2005; Miller and Cleaves, 2006; Kasting, 2009; Russell *et al.*, 2010, 2014; Grosch and Hazen, 2015; Saladino *et al.*, 2016; Sojo *et al.*, 2016).

As is the case still today, porous carbonate edifices developed where alkaline submarine springs liberated their warm exhalation rich in minerals and electron donors, such as H_2_, CH_4_, methanol, and other short-chain hydrocarbons and formates (Kelley *et al.*, 2005; Schrenk *et al.*, 2013; Olah *et al.*, 2017). Hot, acidic hydrothermal fluids also produced porous, “beehive-like” structures, rich in Fe and Mg minerals (Russell and Hall, 1997; Martin and Russell, 2003). The thermodynamic driving force came from the chemical potential of the gases discharged by the vents (Russell *et al.*, 2013). The interconnected micrometer-scale pore spaces in the rock matrix worked as efficient miniature chemical reactors, affording the means to confine, fixate, and enrich across temperature gradients; they also included Fe-, Ni-, and S-bearing minerals that could act as organic catalysts (Hazen and Sverjensky, 2010; Sleep *et al.*, 2011; Deamer and Georgiou, 2015; Konn *et al.*, 2015; Sojo *et al.*, 2016; Olah *et al.*, 2017). The sheer scale and activity of this planet-wide experiment in organic synthesis cannot be compared with the modest levels we see at present: Earth's entire crust was giving birth.

Summarizing, the time to sequester most of the initially hot, mainly CO_2_ atmosphere, liberating enough CH_4_ to compensate for the rapidly diminishing greenhouse forcing of CO_2_, is estimated to be of the order of a hundred million years (Zahnle *et al.*, 2007). Toward the end of this process, by ∼4.4 Ga ago, the ocean waters surrounding the innumerable submarine vents spewing out a rich cocktail of reduced compounds could have attained temperatures less than 80°C (Zahnle *et al.*, 2007; Sleep, 2010). This is important because this value can be considered as an upper limit for the survival of complex organic molecules (Larralde *et al.*, 1995; Miller and Lazcano, 1995).

An enormous chasm lies between molecules and cells, and we do not understand how it was bridged. It is not the case that, once we had an interesting mix of organics, cellular organization took care of itself. This does not happen in the laboratory and most surely did not on early Earth (Schrum *et al.*, 2010). We must, therefore, accept the need for an extended phase (perhaps a few million years—or more—we will never be sure) of prebiotic chemical evolution during which the various molecular building blocks generated and associated, underpinned by replication, to gradually progress from elements to system (Lazcano and Miller, 1996; Joyce, 2002; Orgel, 2004; Harold, 2014). We can perhaps call this a period of converging prebiotic chemistry.

The first viable protocells—probably endolithic autotrophs exhibiting the rudiments of autonomy, energy conversion, and reproduction, but lacking much of the complexity of modern-day archaea—could have relied on physicochemical attributes of the vents' porous network and circulating fluids for many of their functions (Russell and Arndt, 2005). To be able to disperse and settle in other environments, whether reached through open water or the subsurface, along fractures and fault zones, likely required a higher degree of sophistication, including proper membranes.

We do not know exactly when life appeared on our planet, how much it was helped along by the delivery of exogenous matter (Chyba and Sagan, 1992; Thomas *et al.*, 2006; McKay, 2010), or affected by subsequent large meteoritic impacts (Sleep *et al.*, 1989; Zahnle *et al.*, 2007; Marchi *et al.*, 2014). However, a most plausible first window of opportunity (but not the only one) is the one described here: first, because it provided necessary organic ingredients and the microscale physicochemical incubation niches that could have been conducive to life's origin (Saladino *et al.*, 2016; Sojo *et al.*, 2016), and second, because it was multiplied a million-fold over the global oceanic crust, increasing enormously the chances for eventually achieving organisms.

Further evolution of the mantle gradually resulted in the first emerged landmasses (Hawkesworth and Kemp, 2006; Arndt and Nisbet, 2012; Grosch and Hazen, 2015), adding subaerial hydrothermal vents to the list of potential environments for the origin of life (Deamer and Georgiou, 2015). However, the elevated UV dose from the young Sun, coupled with the likely absence of an ozone layer in the primitive atmosphere, could have posed serious problems for the long-term accumulation and chemical evolution of exposed prebiotic compounds on early Earth (and Mars) (Cleaves and Miller, 1998). Protection from UV radiation is another motivation for proposing an origin of life in submarine environments.

As Earth's geothermal engine slowly wound down, so did the number and activity of hydrothermal sites. The associated reduction in atmospheric CH_4_ injection was compensated by a progressive increase in Sun's luminosity. In general, warm ocean conditions prevailed for hundreds of millions of years (Hren *et al.*, 2009), although it is probable that Earth's surface may have experienced a number of cold spells (Ashkenazy *et al.*, 2013), as well as several major impacts (Bada *et al.*, 1994). During this period, microbes diversified, achieved higher degrees of functional complexity, and proceeded to colonize all surface and subsurface habitats available to them. As they spread, microorganisms developed an ever more important capacity to influence environments and affect the regulation of planetary feedback mechanisms, two factors that may have contributed greatly to life's enduring persistence on Earth (Chopra and Lineweaver, 2016).

#### 3.2.2. Young Mars

It is likely that by 4.45 Ga ago, early Mars also had developed a global 500 K ocean (or large bodies of water) enveloped in an ∼100 bar, mostly CO_2_ atmosphere (Elkins-Tanton, 2011). But Mars was much further away from the young Sun than its siblings. It was also smaller and, therefore, lost heat faster. The solar flux reaching Mars at 1.52 AU is lower than that illuminating Earth (currently 1365 W/m^2^) by a factor ${ \left( {1.00 \;{ \rm{AU}} / 1.52 \;{ \rm{AU}}} \right) ^2} = 0.43$ Assuming a solar luminosity 70% that of today, the mean annual energy reaching Mars 4.4 Ga ago would have been in the order of $S = 0.70 \;  \times  \;0.43 \;  \times  \;1365$ W/m^2^$= 411$ W/m^2^. Plugging this number into the planetary energy balance equation, we get

\begin{align*}
\sigma T_e^4 = \frac { S }  { 4 } \left( { 1 - A } \right) ;
\end{align*}

where *T_e_* is the effective black body radiation temperature as if the planet had no atmosphere, *S* is the solar flux, $\sigma$ is the Stefan–Boltzmann constant ($5.67 \;  \times  {10^{ - 8}}$ W/m^2^/K^4^), and *A* is the albedo; assuming a very low, cloud-free 0.05 value for a water-covered early Mars (currently it is about 0.33 for Earth and 0.17 for Mars); we calculate an effective temperature of ${T_e} =$204 K. If instead we consider $A = 0.30$, we obtain ${T_e} =$189 K. These results suggest that, in the absence of other inputs, Mars would have quickly frozen over (Fairén *et al*., 2012). However, as on Earth, we can expect that very active subsurface hydrothermal processes driven by internal heat may have helped raise surface temperature by releasing CH_4_ and other gases (Pavlov *et al.*, 2000; Oze and Sharma, 2005; Schulte *et al.*, 2006).

To help put this into perspective, we consider again the planetary energy balance equation. The solar flux reaching early Earth 4.4 Ga ago was $$S = 0.70 \;  \times  \;1365$$ W/m^2^ $$ = 955$$ W/m^2^. This implies a $${T_e} =$$252 K (for $$A =$$0.05) to $${T_e} =$$233 K (for $$A =$$0.30). If we consider the generally warm temperature of the terrestrial ocean (at least at the rock–water interface) as derived from isotopic measurements carried out on ancient rocks, we have to conclude that the atmosphere (whatever CO_2_ was left at the time, plus H_2_O and CH_4_) provided (as a minimum) 50 K (for $$A =$$0.05) to 70 K (for $$A =$$0.30) increase over *T_e_* by greenhouse trapping (this value is 33 K for present-day Earth).

If we examine what could have happened if Mars' internal engine and mantle had made a similar greenhouse contribution, we can see that, even assuming an increase of 70 K, the average surface temperature would have hovered around water's freezing point (*e.g.*, 204 + 70 K = 274 K). Hence, for a good part of its early history, Mars could have perhaps looked like a colder version of present-day Iceland—gelid on top, heated from below. However, the likelihood of a cold surface scenario does not constitute a serious obstacle for the possible appearance of life, as extensive subglacial, submerged, and emerged volcanic/hydrothermal activity would have resulted in numerous liquid water-rich settings (Warner and Farmer, 2010; Cousins and Crawford, 2011). The right mixture of ingredients, temperature and chemical gradients, organic molecule transport, concentration, and fixation processes could have been found just as well in a plethora of terrestrial submarine vents as in a multitude of vents under (maybe) top-frozen martian bodies of water (Westall *et al.*, 2013; Russell *et al.*, 2014).

#### 3.2.3. Young Venus

How was early Venus any different from Earth? Both had a similar beginning, almost the same size (although very different rotation rates), and roughly equal internal and surface environments. There is the detail of the Moon formation impact, but this happened so early that it can probably be ignored for the sake of this discussion. The mean annual solar energy reaching young Venus would have been 1825 W/m^2^ (roughly in the middle between Earth's present-day 1365 W/m^2^ and Venus' current 2607 W/m^2^). We calculate an effective temperature of ${T_e} =$295 K, quite warm (this is $\sim$20 K more than our planet's current *T_e_*) but not enough to prevent the initially hot atmosphere from cooling. If instead we consider $A =$0.30, we obtain ${T_e} =$273 K.

If we assume that the first stage of crustal–atmospheric evolution on Venus proceeded more or less as it did on Earth, much CO_2_ would have been rapidly fixed in the planet's interior through carbonation and subduction of the oceanic crust. Meanwhile, serpentinization in the shallow ocean crust would have produced H_2_ that reacted with CO_2_ and CO to release CH_4_ and other simple organic molecules. However, whereas the additional heat input provided by the gradual buildup of atmospheric CH_4_ was helpful on Earth, this was not the case on Venus.

If as before we raise temperature on Venus by 50 to 70 K, this would imply ocean temperatures of the order of 70°C, for example, 295 $+$ 50 K $=$ 345 K (for $A =$0.05). This simple calculation would suggest that a potentially brief early Venusian ocean could have been uncomfortably warm for the stability of prebiotic chemical products. Second, we would need to consider the surface environment evolution as the production of CH_4_ waned and solar luminosity increased. The timing of the relative contributions is important. To obtain a coherent scenario would require careful modeling, considering a number of possible variations that may help us to constrain what could have happened and when—this exceeds the scope of this article. The question is still open regarding the possibility that young Venus could have harbored (for some yet-to-be-determined period) surface conditions allowing life to (perhaps) gain a fleeting foothold (Schulze-Makuch *et al.*, 2013).

### 3.3. Lessons for ExoMars: when and where?

Hopefully having made the case that conditions for the appearance of microbes on early Mars were similarly favorable as on our planet, it remains for us to examine how likely it is that we may find evidence, or at least some clues, of their presence. Here we move into the realm of that word: *habitability*. Originally defined as a planet's potential to hold life of any kind, a more “binary” definition was introduced by Cockell *et al.* (2016): an environment is habitable if capable of supporting the activity of at least one known organism—yes or no (although in reality microbial colonies in nature are almost always multispecies). Considering the need to find landing sites suitable for pursuing our mission's science, we should establish a metric to inform us whether, how much, when, and how long a place had the capacity to host and nurture cells—the only living machine we are aware of.

An interesting approach is that used in the domain of planetary protection. Minimum temperature and water activity thresholds have been identified below which even the hardiest known terrestrial microorganisms cannot replicate. These parameters are used to classify areas of present Mars in terms of their potential to become habitats for spacecraft delivered Earth microorganisms (Kminek and Rummel, 2015; Kminek *et al.*, 2016; Rettberg *et al.*, 2016). We, however, require constraints that are able to boost our confidence that microbes could have thrived in the past (Stoker *et al.*, 2010; McLoughlin and Grosch, 2015), a dynamic past (*e.g.*, impacts and obliquity cycles) for which we can only infer conditions on the basis of patchy geological information and theories.

Another important issue is scale. Earth regions that at first glance may seem barren, such as the Atacama Desert or the Antarctic Dry Valleys, include numerous localized pockets where microbes toil away (Pointing *et al.*, 2009; Crits-Christoph *et al.*, 2013; Azua-Bustos *et al.*, 2015). However, we can agree that neither of those locations would be our preferred target to look for biosignatures on our planet, particularly if we had to choose on the basis of orbital data. The reason is that both places seem drab and devoid of liquid water when observed from far away.

On Earth, the vast majority of organisms live, evolve, and die without leaving long-lasting traces of their existence. Not surprisingly, our fossil record is dominated by species that inhabited environments with high preservation potential, where sediment accumulation led to rapid burial, for example, in or around lakes, rivers, swamps, and marine basins. Organisms that were soft bodied or occurred in ephemeral habitats are seldom preserved. Species that existed over a broad area have a higher probability of being found than those that were rare or geographically restricted (Hull *et al.*, 2015); the same applies to landed planetary missions.

Stating that a place was once “habitable” does not help us much when designing a search-for-life mission. We would prefer to know how much more likely a location was than another to have been extensively colonized for long periods. While recognizing that what matters to microorganisms takes place at minute scales, our ability to find their traces, which depends strongly on their dissemination, does not. Hence, when it comes to boosting our chances of detecting biosignatures, scale and preservation need to be considered together. We, therefore, propose to categorize a candidate landing site's habitability in terms of the extent and frequency of liquid water lateral connectivity between the potential (micro) habitats. For example, although both would have been habitable, a single, short-lived meandering channel would constitute a less appealing target than a network of interconnected lakes having undergone numerous inundation episodes (wetter for longer).

Despite certain obscurities and yet unanswered questions, life seems to have appeared on our planet as soon as the environment allowed it, sometime between 4.4 and 3.8 Ga ago. It then continued onward more or less hampered by large impacts, a few of which could have done away with most exposed and shallow subsurface organisms (Thomas *et al.*, 2006). Although colder, we also postulate that conditions existed for the possible emergence of life on Mars (Solomon *et al.*, 2005; McKay, 2010; Strasdeit, 2010; Yung *et al.*, 2010).

**FIG. 2.**
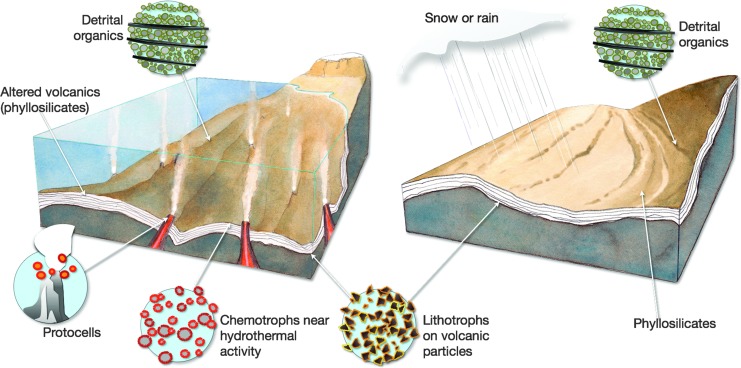
Diagram showing plausible Mars habitable environments during the Early- to Middle-Noachian. Some of these settings may have been active long enough to witness the appearance of life (especially in the case of long-term hydrothermal activity); others could have hosted already flourishing microorganisms.

On Earth, microbial life quickly became a global phenomenon. Fueled by a young planet's internal heat, a similar explosive process could have occurred early in the history of Mars. However, the availability of transport paths between liquid water-rich environments proceeded very differently on the two planets. Sometime during the late Noachian, martian surface habitats gradually became more isolated; their lateral connectivity started to dwindle and eventually disappeared (Westall *et al.*, 2013, 2015a) (Fig. 1E). This situation could be described as “punctuated” habitability. As surface conditions deteriorated, potential microbes could have found refuge in subterranean environments (Michalski *et al.*, 2013a). Occasionally, impact-formed hydrothermal systems would have resulted in transient liquid water becoming available close to the surface, even if the martian climate was cold (Rathbun and Squyres, 2002). But it does not necessarily follow that these later habitats could have been colonized (Cockell *et al.*, 2012). We, therefore, conclude that, to maximize our chances of finding signs of past life on Mars, we must target the “sweet spot” in Mars' geological history, the one with the highest lateral water connectivity—the early Noachian—and look for large areas preserving evidence of prolonged, low-energy, water-rich environments, the type of habitat that would have been able to receive, host, and propagate microorganisms (Fig. 2).

The absence of plate tectonics on Mars (van Thienen *et al.*, 2004) increases the probability that rapidly buried, ancient sedimentary rocks (possibly hosting microorganism biosignatures) may have been spared thermal alteration and been shielded from ionizing radiation damage until uncovered by aeolian erosion relatively recently (Malin and Edgett, 2000).

## 4. Biosignatures: Which and How Reliable?

The main challenge for any search-for-life mission consists in determining whether a candidate observation (or better yet, a collection of observations) can be uniquely attributed to the action of biology (Cady and Noffke, 2009).

We next discuss a large list of measurable biosignatures. The science payload on board the ExoMars rover (Section 6) can only address a subset of these.

Microorganism biosignatures can be grouped into three broad categories (Cady *et al.*, 2003) as follows: (1) cellular fossils that preserve organic remains of microbes and their extracellular matrices; studying them typically requires complex sample preparation and high-resolution instruments not currently available on landed space missions (Westall *et al.*, 2011a); (2) bioinfluenced fabrics and sedimentary structures (Westall, 2008, 2012; Davies *et al.*, 2016), which provide a macroscale imprint of the presence of microbial biofilms that can be more readily identified, for example, laminated stromatolites; (3) organic chemofossils preserved in the geological record (Parnell *et al.*, 2007; Summons *et al.*, 2008) that can be either primary biomolecules or diagenetically altered compounds known as biomarkers.

### 4.1. Morphological biosignatures

In terrestrial marine (and other wet) environments, benthic microorganisms (*e.g.*, those living in the seabed) form biofilms, highly organized microbial communities that are able to affect the accumulation of detrital sediments. Particle binding, biostabilization, baffling, and trapping by biofilms can result in macroscopic edifices amenable to be recognized and studied with rover cameras and close-up imagers. These are collectively known as microbially induced sedimentary structures (MISS) (Noffke and Awramik, 2011; Noffke *et al.*, 2013; Davies *et al.*, 2016). In cases where sediment precipitation occurs in a repetitive manner, multilayer constructions can ensue; for example, stromatolites constitute essential beacons of information, recording snapshots of microbial communities and environments throughout Earth's history (Allwood *et al.*, 2006, 2009, 2013). MISS and stromatolites stem from the cooperative action of microbes, in particular phototrophs produce large amounts of extracellular polymeric substances in the biofilm. If the biofilm covers a large enough area experiencing similar conditions, often multiple organosedimentary structures can arise in regularly spaced groups—see, for example, Figure 1 in the work of Allwood *et al.* (2006). Nevertheless, Davies *et al.* (2016) noted that MISS should be treated with caution as they are a subset of “sedimentary surface textures” that include those of abiotic origin.

But the presence of microbes does not always lead to the emergence of noticeable macroscale biosedimentary formations. An example of a less conspicuous expression is the layering found in some typical early Earth volcanic lithic environments, where organisms have colonized the surface of ashfall particles, creating visible, carbon-rich, black biofilms on various sediment horizons (Westall *et al.*, 2011b).

The primordial types of microorganisms that could have existed on early Mars would have been tiny and of the order of a micron to a few microns in size. The individual cells would be too small to distinguish. However, as on Earth, their permineralized or compressed microbial colonies and biofilms would be much larger. Traces of these features may be preserved on martian rocks as mineral-replaced structures and/or as carbonaceous remains trapped in sediments encased in mineral cement. Rover cameras and, in particular, high-resolution close-up imagers would be able to investigate many candidate microbialites similar to terrestrial thrombolites, stromatolites, layered biofilms, and abiotic/biotic organic particles and laminae (Westall, 2008; Westall *et al.*, 2015b; Ruff and Farmer, 2016). Nevertheless, in more than 20 years of Mars surface exploration, and after having studied numerous examples of laminated sedimentary structures, there have been no claims gathering widespread support for the presence of biomediated structures.

### 4.2. Chemical biosignatures

Most of Earth's biological matter exists in the form of carbonaceous macromolecules stored within layered sedimentary rocks, which are orders of magnitude more abundant than that in living beings (Summons *et al.*, 2011). If life existed on ancient Mars, its remains may also have accumulated in extensive, organic-rich sedimentary deposits.

When considering molecular biosignatures, the first obvious set of targets is the ensemble of primary biomolecules associated with active microorganisms, such as amino acids, proteins, nucleic acids, carbohydrates, some pigments, and intermediary metabolites. Detecting the presence of these compounds in high abundance would be diagnostic of extant life, but unfortunately they degrade quickly once microbes die. Lipids and other structural biopolymers, however, are biologically essential components (*e.g.*, of cell membranes) known to be stable for billions of years when buried (Brocks, 1999; Georgiou and Deamer, 2014). It is the recalcitrant hydrocarbon backbone that is responsible for the high-preservation potential of lipid-derived biomarkers relative to that of other biomolecules (Eigenbrode, 2008).

Along the path from primary compound to molecular fossil, all biological materials undergo *in situ* chemical reactions dictated by the circumstances of the source organisms' transport, deposition, entombment, and post-depositional conditions. The end product of diagenesis is macromolecular organic matter, which, through the loss of superficial hydrophilic functional groups, slowly degrades into the solvent-insoluble form of fossil carbonaceous matter called kerogen, but not all information is lost. The heterogeneous chemical structure of the kerogen matrix can preserve patterns and distribution diagnostic of biosynthetic pathways. Kerogen also possesses molecular sieve properties allowing it to retain diagenetically altered biomolecules (Tissot and Welte, 2013).

Besides the direct recognition of biomolecules and/or their degradation products, other characteristics of bioorganic compounds include the following (Summons *et al.*, 2008, 2011):

#### 4.2.1. Isomerism selectivity

- *Enantiomeric excess:* In the case of chiral molecules (those that can exist in either of two nonidentical mirror image structures known as enantiomers), life forms synthesize exclusively one enantiomer, for example, left-handed amino acids (l-amino acids) to build proteins and right-handed ribose (d-ribose) for sugars and the sugars within ribonucleic acid (RNA) and deoxyribonucleic acid (DNA). Opposite enantiomers (d-amino acids and l-ribose) are neither utilized in proteins nor in the genetic material RNA and DNA. The use of pure chiral building blocks is considered a general molecular property of life.
  When an organism dies and its biochemicals are released into the environment, the enantiomeric enrichment in the molecular building blocks may or may not endure. Over time, the action of a number of physicochemical processes can result in racemization, that is, the pathway that ultimately leads to an equal mixture of the two enantiomers, called a racemate. How fast this racemization of life's chiral molecular building blocks happens depends on the intensity (dose, temperature, pH, etc.) and duration (continuous, cyclical, pulsed, etc.) of the perturbing action, and on the compound's chemical stability, particularly of the bonds surrounding the chiral center.
- *Diastereoisomeric preference:* Just as biologically produced amino acids (single chiral center) occur preferentially as one enantiomer, other products with multiple chiral centers, such as some organic acids, isoprenoids, tocopherol (vitamin E), chlorophyll, and sugars, are also biosynthesized as a unique stereoisomer.
- *Structural isomer preference:* For even more complex organic compounds (*e.g.*, with multiple ring systems and degrees of unsaturation) where many structure or constitutional isomers are possible, life tends to use a limited subset of all the isomers that would be chemically feasible.

Although amino acid homochirality can be an important biosignature, recent measurements of l-enantiomeric excess values for some conglomerate-forming α-H proteinogenic amino acids on fragments of the Tagish Lake meteorite (Glavin *et al.*, 2012) show that nonbiological processes could also lead to significant enantioenrichment for some amino acids. It is, therefore, important to perform a holistic chemical interpretation, evaluating a number of compounds and their relationships.

#### 4.2.2. Molecular weight fingerprints

- *Uneven distribution patterns of clusters (C number, concentration, and δ^13^C) of structurally related compounds:* Many important biochemicals exist in discrete molecular weight ranges (*e.g.*, C_14_–C_20_ lipid fatty acids). For this reason, the molecular weight distribution of biologically derived matter exhibits clustering; it is concentrated in discrete clumps corresponding to the various life-specialized families of molecules (Summons *et al.*, 2008). This is in contrast to the molecular weight distribution for cosmic organics (Ehrenfreund and Charnley, 2000; Ehrenfreund and Cami, 2010): the relative abundance for abiotic volatiles is uniform and drops off as the carbon number increases.
- *Repeating constitutional subunits:* Many biological products (*e.g.*, proteins and nucleic acids) are synthesized from a limited number of simpler units. This can leave an identifiable molecular weight signature even in fragments recovered from highly derived products, such as petroleum. For example, in the case of material containing fossil lipids, we would expect to find a predominance of even-carbon numbered fatty acids (C_14_, C_16_, C_18_, C_20_). This is because the enzymes synthesizing fatty acids attach two carbon atoms at a time (in C_2_H_4_ subunits) to the growing chain. Other classes of biomolecules can also exhibit characteristic carbon chain length patterns, for example, C_15_, C_20_, and C_25_ for acyclic isoprenoids constructed using repeating C_5_H_10_ blocks.
- *Systematic isotopic ordering at molecular and group level:* Biological molecule building blocks, in particular some functional groups, can show significant differences in their degree of ^13^C incorporation relative to ^12^C. The “repeating subunit” conformation of biomolecules can result in an observable isotopic ordering in the molecular fingerprint.

#### 4.2.3. Bulk isotopic fractionation

The isotopic fractionation of stable elements such as C, H, O, N, S, and Fe can be used as a signature to recognize the action of biological pathways. Although the qualitative chemical behavior of the light and the heavy isotope is similar, the difference in mass can result in dissimilar bond strength and reaction rates. Thus, the isotopic discrimination associated with organic biosynthesis (which alters the natural equilibrium between C isotopes in favor of the lighter variant) is principally responsible for determining the ^13^C/^12^C ratios in terrestrial organic and inorganic crustal reservoirs.

Although interesting, we do not consider bulk isotopic fractionation a robust biosignature when applied to locations or epochs for which we have scant knowledge of sources and sinks. In the specific case of carbon, ^13^C/^12^C ratios may serve as reliable biosignatures for past or present life only if the key components of the C-cycling system (applicable at the time of deposition and since then) are well constrained (Summons *et al.*, 2011). This is certainly not the case for Mars, and one can also wonder to what extent we are sure about our own past carbon dynamics when analyzing very ancient samples.

Despite the mentioned reservations, we are willing to include bulk isotopic fractionation in this list, but with the caveat that it should be used in association with other, less indirect, biosignatures.

### 4.3. Importance of geological context for boosting biosignature confidence

Demonstrating that a sample has been obtained from a geological setting that possesses long-duration aqueous attributes that could have allowed hosting and propagating microorganisms would help to increase substantially the confidence of any potential biosignature claim.

This characterization of geological context begins early, with landing site selection, as investigators canvas candidate locations searching for those that best fit the mission's scientific objectives. However, experience has shown again and again that, when it comes to Mars, often what we thought we understood from orbit is found to have concealed a few surprises once we examine things at close range.

When studying rocks, it is important to distinguish syngenetic from postgenetic features. The former relate to the original deposit and its formation (aggradational) environment (aqueous, aeolian, volcanic, etc.), whereas subsequent (degradation) processes are responsible for the latter. Postgenetic processes may act relatively quickly after rock formation, for instance, diagenetic changes to sediments deposited in water or to volcanic rocks extruded into water. They may also occur millions of years afterward because of major environmental changes or external events, for example, impacts, later volcanic/hydrothermal action, subsurface fluid migration, or mass wasting/erosional/weathering phenomena.

Detailed visual and mineralogical studies are fundamental for correctly interpreting rock type and mode of formation. Accurately characterizing stratigraphy, structure, textural relationships, and grain mineral matrix properties allows to distinguish, for example, *in situ* brecciation, transport by physical mass wasting, glacial, or fluvial processes. Especially grain size, shape, and size distribution can teach us much about transport mechanisms and their duration. Well-rounded clasts often indicate extended movement, or, alternatively, deposition in an agitated environment with much grain-to-grain contact and erosion. Angular clasts usually signal deposition close to the source of the clasts, although supraglacial and englacial debris can be transported for kilometers with no substantial rounding. Finer grained sediments are typically associated with distal deposition (*i.e.*, longer transport) or with the erosion of originally fine-grained, friable material. An example of this kind of textural analysis is the sedimentological study of the conglomerates at Bradbury Rise, in Gale Crater, Mars, that showed fluvial transport at the time of deposition, ∼3.6 Ga ago (Williams *et al.*, 2013). The finely laminated mudstones found in Gale Crater have been interpreted as distal deposits of sediment plumes discharging into a body of standing water during a period lasting in the order of 100 to 10,000 years in the early Hesperian (Grotzinger *et al.*, 2015). Mudstones could constitute an interesting target for the ExoMars rover, as would many clays.

### 4.4. Life's decision points

As a species, humans are largely visually orientated. We tend to believe in what we can see, but when it comes to tiny microorganisms, images alone can be deceiving (García-Ruiz *et al.*, 2002, 2003, 2009). So what would constitute an ideal positive detection of life on Mars, the *non plus ultra*? Perhaps the following: (1) Discover a group of candidate biosedimentary structures embedded in a congruent geological landscape, that is, an environment that demonstrably possessed attributes conducive to the prosperity of microbial communities, for example, a long-lived, low-energy, shallow aqueous, or hydrothermal setting experiencing frequent fine sediment deposition. (2) Zoom in at microorganism scale to discern individual fossilized microbial cells, colonies, or biofilms and their extracellular matrix. (3) Extract and analyze carbonaceous matter from the putative colonies/biofilm and obtain chemical indicators that confirm their biogenicity. Unfortunately, this we cannot achieve because the mentioned scenario requires an unlikely convergence of deposition, preservation, and exhumation conditions coupled with a payload able to prepare and analyze samples as in an Earth laboratory, something still not possible with our robotic landed mission's capabilities.

Is there a pragmatic set of robust measurements that could provide proof of life? Better yet, can we devise a scale or scoring system to help us quantify how confident (or otherwise) we have a right to be? Here, we propose one such scheme, which is not to be taken literally, but to stimulate discussion and hopefully lead to an improved version. ExoMars, and other life-seeking missions, would benefit greatly from such a tool.

**FIG. 3.**
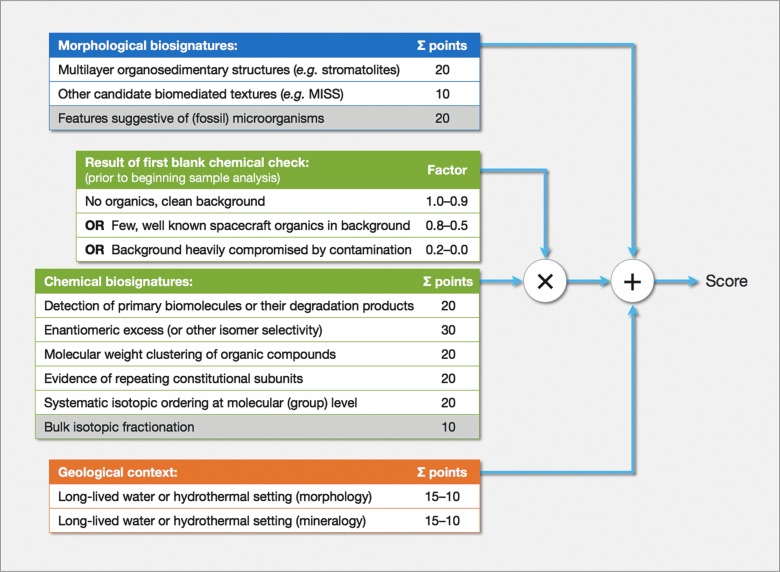
ExoMars Biosignature Score: A possible system to assign a confidence value (the score) to a group of robust observations aiming at establishing whether a location hosted life. We have indicated with a gray background the biosignatures that the ExoMars rover payload is not equipped to assess.

Figure 3 presents a possible system for assigning a confidence value (the score) to a group of observations with the intent to establish whether a location on Mars (or elsewhere) hosted microbial life, past or present. We have called this the *ExoMars Biosignature Score* because it is being developed while preparing for this mission; however, the list of biosignatures included is rather complete and encompasses more than what ExoMars will be able to assess.

The ExoMars rover can search for two broad classes of biosignatures: (1) morphological: textural information preserved on outcrops, rocks, and collected samples and (2) biochemical: in the form of bioorganic compounds and their degradation products. The rover is also capable of exploring the landing site and establishing the geological environment at the time of deposition and its subsequent evolution.

The biosignatures that the Pasteur payload cannot address are (1) visual recognition of individual organism microfossils, which is only achievable on Earth with very high-magnification instruments, for example, electron microscopy conducted on thin-section, acid-etched samples and (2) bulk isotope excursions, which we claim are not as robust a diagnostic as others.

Within the available resource envelope, the science team tried to implement the techniques we believed could, when used in a combined manner, give us the best chance to achieve a (potential) positive detection.

Please note that Figure 3 does not include morphological changes with time, movement, or experiments designed to elicit active metabolic responses (as in Viking). These “more dynamic” expressions of possible present life would not be easy to verify. They can be taken into account in case a later mission is designed to pursue them.

The individual findings shown in Figure 3 (reflecting the positive outcome of a given investigation, *i.e.,* a verified biosignature) are grouped into three major categories: (1) morphological biosignatures, (2) chemical biosignatures, and (3) geological context information. The latter does not include biosignatures, but can bolster the claims of other measurements. The numbers on the right correspond to the score afforded to each “confirmed biosignature,” reflecting their relative importance. For example, detecting patent (*i.e.*, significantly larger than for meteorites) enantiomer excess in organic matter recovered from Mars samples would be strongly suggestive of a biological origin; hence, verifying this has a larger associated value than, say, establishing that liquid water was available at the site.

The validity of all chemical biosignatures is modulated by a multiplicative “quality factor.” This factor depends on the outcome of a blank chemical check when using a suitably characterized material made to transit through all mission elements coming into contact with martian samples. This test must be conducted before commencing any chemical investigations, analytic or spectroscopic. Depending on the results of the blank check, one could have (1) a chemical background devoid of organic contamination, in which case the factor can be high (1.0–0.9, according to characteristics of the floor level). (2) Some well-understood spacecraft contamination with possible effects ranging from modest to severe, depending on how much its chemical fragment background masks potential true biosignatures; this could result in factor values between 0.8 (for a relatively benign case) and 0.5 (when the effect is more critical). It is worth noting that the level of contamination may change during the course of a mission in terms of quality (*i.e.*, type of molecules) and quantity. Therefore, it would be advisable to carry sufficient blanks to repeat this test, as the analytical conditions could improve. (3) A chemical background heavily compromised by terrestrial contamination, for example, if the instruments were exposed to Earth's open-air environment before launch. Unless there is the means to return the spacecraft to pristine conditions on the surface of Mars, this would seriously affect the mission's ability to identify chemical biosignatures. The corresponding factor is, therefore, very low (0.2–0.0). Regarding geological context—not a direct biosignature—we propose a restricted range of values, higher or lower depending on the frequency and extension of the liquid water environment's lateral connectivity.

Having performed a complete set of investigations at one location, we would first tally up the points for each group of “biosignatures.” The score for chemical biosignatures is multiplied by the previously discussed quality factor. Finally, all contributions are summed up to compute the final score. On the basis of its value, one would conclude (1) (score ≥ 100) the ensemble of results obtained proves there was life at this site; (2) (50 ≤ score < 100) some observations are consistent with a possible biological presence, but are not conclusive; (3) (score < 50) insufficient evidence.

A closer examination of Figure 3 reveals that, if we could tick all possible biosignatures, assigning maximum points with a perfect chemical background, the score would be 200 (170 if we only consider what ExoMars can detect), whereas we claim we only need a value of 100 to establish that there was/is life. This is so to indicate that it is not necessary to verify all possible biosignatures, but that it is mandatory to provide evidence that a few of the principal biosignatures are indeed demonstrated. Chemical biosignatures are awarded a higher importance, and rightfully so. They provide “more direct” evidence of biogenicity than the other categories for which bioinfluence is “inferred.”

### 4.5. Examples using the ExoMars Biosignature Score

The proposed system needs to be validated with suitable tests. It is not easy to find documented instances where the entire set of measurements in Figure 3 has been performed on samples obtained at one location. Often, the type of analysis reported mirrors the main expertise of the team writing the article, for example, geological interpretation, spectral composition, or analytical chemistry. We believe a holistic approach that covers all aspects (morphological biosignatures, molecular biosignatures, and geological context) is necessary to arrive to an informed decision concerning the possibility of life. Hereafter we discuss four cases: two of them are studies of early Earth samples, the others are of Mars material.

#### 4.5.1. Kitty's Gap, N.W. Australia

In this section, we produce a score for the 3.446-Ga-old Kitty's Gap chert collected in the Pilbara Craton, N.W. Australia (Westall *et al.*, 2006a, 2011a, 2015b; Bost *et al.*, 2013). This formation consists of volcanic sediments deposited in a coastal mudflat environment, a relevant analogue for shallow water settings on Noachian Mars.

- *Geological context:* The Kitty's Gap chert formed in a mudflat/infilling tidal channel setting. The observed black and gray laminated sediments consist of millimeter- to centimeter-thick layers of different mineral grain sizes; coarser layers are light, whereas finer, silt- to clay-sized material is much darker. Silica-saturated seawater and silica-rich fluids from another local hydrothermal source caused a rapid lithification of sediments and microorganisms more or less contemporaneous with their deposition. Analyses with a few ExoMars representative instruments (visual, IR, and Raman) confirmed the sedimentary nature of the rock and revealed the presence of water-containing minerals and disordered carbonaceous matter. We accord 30 points for establishing the habitable nature of the water setting, both morphologically and through mineralogical analysis.
- *Morphological biosignatures:* No macroscopic traces of fossilized life are observed in association with the specimen. Whereas distinct layers are visible, they cannot be attributed to microbial formation; they record multiple stages in the deposition process. The sample was found to host small (<1 μm in size) microorganisms that formed colonies around volcanic particle surfaces. The preserved microbial communities are dominated by coccoids, but some locally transported filaments suggest the possibility that photosynthetic mat fragments, perhaps broken up by wave or tidal activity, were incorporated into the sediments. We assign 20 points for the identification of fossil microorganisms in various stages of development, including division and death.
- *Chemical biosignatures:* Bulk carbon concentrations in the sample range from 0.01 to 0.02 wt %. The carbonaceous fraction was found to be mature kerogen in accordance with the low-grade metamorphic history of the rock. No detailed analytical inventory of the organic species and their properties was conducted on this sample. However, analysis of the organic carbon by stepped combustion documents clumped isotopic signatures with an average δ^13^C value of −27.8‰ to −25.9‰, in principle consistent with microbial fractionation of carbon. We can only assign 10 points.
- *Discussion:* A score of 60 is near the minimum for considering that a sample may record traces of a possible biological presence, but 20 points come from the recognition of fossil microorganisms that would not be feasible with typical spacecraft (*e.g.*, ExoMars) instrumentation. Although the carbon isotope composition is suggestive of the possible action of life, a more detailed, MOMA-like chemical characterization of the organic matter would be necessary to increase the overall score.

#### 4.5.2. Josefsdal Chert, Barberton, South Africa

We next assign a score to 3.333-Ga-old Josefsdal Chert samples from the Barberton Greenstone Belt, South Africa, which have been subjected to a more complete battery of chemical analyses than the Kitty's Gap rocks (Westall *et al.*, 2006b, 2011b, 2015a, 2015b).

- *Geological context:* The Josesfdal Chert formation consists of silt- to sand-sized volcanic sediments that were deposited in an upper offshore to upper shoreface setting (*i.e.*, from some tens of meters water depth to exposed beach), as evidenced by sedimentary structures ranging from low-amplitude dunes to wave ripples. The depositional environment was continuously bathed, to a greater or lesser extent, by warm hydrothermal fluids. This is documented by intrusions of silica-rich fluids parallel to sediment layering, by intrusions causing soft sediment deformation, by early diagenetic silicification, as well as by characteristic geochemical signatures (presence of diagnostic trace elements, Cu, Fe, Zn, etc.). Importantly, all the volcanic clasts were altered to phyllosilicate before silicification, supporting the interpretation of deposition in water.
  Measurements with ExoMars representative instruments (visual, IR, and Raman) confirmed the sedimentary nature of the rocks and established the presence of water-containing minerals and disordered carbonaceous matter. The Josefsdal Chert volcanic sediments can be attributed 30 points because they demonstrate prolonged habitable conditions in terms of aqueous environment as deduced from sedimentary structures and mineralogical analysis.
- *Morphological biosignatures:* As with the Kitty's Gap sample, no macroscopic traces of fossilized life can be observed in association with this specimen. At the microscopic scale, however, many recognizable biosignatures exist, ranging from thin biofilms produced by phototrophs at the surfaces of sediment layers to carbonaceous clots created by chemotrophic colonies, either at the surfaces of volcanic particles, as in the Kitty's Gap sediments, or floating in silica-rich hydrothermal fluids. Sediments formed in the vicinity of hydrothermal vents that were colonized particularly extensively by microbial life present a matt black color that is visually distinguishable from sediments experiencing a lesser degree of colonization. We can assign 20 points for the unambiguous identification of fossil microorganisms.
- *Chemical biosignatures:* The total carbon content of this rock is variable, ranging from about 0.01 to (in contrast to the Kitty's Gap sample) 0.5 wt %; the latter was recorded in the already mentioned carbon-rich layers influenced by hydrothermal activity, which can be explained by the fact that hydrothermal fluids are rich in nutrients and can sustain a higher biomass concentration. Raman spectra show that the carbon is mature kerogen, in agreement with the geological age and history of the host rock.
  More detailed analyses with time-of-flight secondary ion mass spectrometry (ToF-SIMS) and sulfur K-edge X-ray absorption near edge spectroscopy allowed the detection of aromatic carbon molecules, such as phenanthrene, anthracene, and thiophene. Although these compounds can also be found in abiotic carbon within carbonaceous chondritic meteorites, the restricted range in their composition is indicative of a biological origin. Repeating molecular subunits are visible in the ToF-SIMS spectra. The carbon isotope ratios measured in bulk by stepped combustion, as well as *in situ*, have an average δ^13^C value of −26.7‰, consistent with microbial fractionation. We can thus attribute 50 points for the verification of molecular weight clustering, repeating constitutional subunits, and bulk isotope fractionation.
- *Discussion:* This rock has been subjected to some of the most sophisticated analytical techniques available today, including synchrotron radiation (Westall *et al.*, 2011b, 2015b). With a total of 100 points, we have a strong body of evidence for the presence of life. However, as for the Kitty's Gap sample, 20 points come from the identification of fossilized microbial cells, colonies, and biofilms/mats when using instruments that are not possible on a typical mission payload. We can conclude, on the basis of suitable habitability and chemical analysis of the organic molecules (which MOMA is also capable of detecting), that had we analyzed this sample with the ExoMars payload (and achieved the same results), we would have scored just 70; this is encouraging, but still insufficient.

The outcome of this and the previous exercise illustrates two points as follows: (1) That the scoring method is tough. To satisfy a naturally skeptical community, we require confirming evidence from a multi-instrument, multidisciplinary approach. (2) That unless samples can be recovered in a very good state of chemical preservation, it will be difficult to demonstrate biogenicity *in situ*. The final verification of a possible life presence may require the analysis of (even the best) samples on Earth.

#### 4.5.3. Martian Meteorite ALH84001

In 1996, David McKay and his colleagues published the first description of possible microbial signatures in extraterrestrial rocks, namely in a meteorite from Mars called ALH84001 (McKay *et al.*, 1996). The subject was so delicate that President Bill Clinton announced the news in a press conference (Statement, 1996). The ensuing interest in the scientific world spurred a huge increase in astrobiological research and, in particular, the study of biosignatures.

- *Geological context:* The precise geological context of the meteorite is not known. The rock is an igneous cumulate, that is, a coarse grained, pyroxene-rich basalt that probably formed at the base of a thick lava flow. Initially dated at about 4.5 Ga, its igneous crystallization age is now placed at 4.09 Ga, during a period of intense bombardment and slightly before the cessation of the Mars global magnetic field (Lapen *et al.*, 2010). ALH84001 is characterized by fractures produced by two shock events, the earliest dating to ∼4.0 Ga ago (McKay *et al.*, 1996). Of interest are flattened, semicircular, 3.94-Ga-old (Borg *et al.*, 1999), Fe- and Mg-zoned carbonates within the fractures. These carbonate globules were likely deposited by low-temperature fluids circulating through the fractures (Gibson *et al.*, 2001). Summarizing, a probable scenario is that the parent rock crystallized and was affected by low-temperature fluids during a period when we expect liquid water to have been available on Mars. The mineralogical information indicates aqueous alteration, but there is no compelling evidence for a long-standing water or hydrothermal setting. We award 10 points.
- *Morphological biosignatures:* The Fe-rich rims of the zoned carbonate deposits consist of aggregates of features having ovoid and elongated morphologies ∼100 nm in length and ranging between 20 and 80 nm in diameter (McKay *et al.*, 1996). McKay *et al.* compared these features to nanobacteria described from terrestrial carbonates. At face value, these aggregates could be awarded a score of 20 as candidate fossil microorganisms, but it appears that they are too small and are more probably corrosion features of the carbonate (Gibson *et al.*, 2001). We prefer not to award any points in this category.
- *Chemical biosignatures:* Although ALH84001 is basically made of coarse-grained lava, polycyclic aromatic hydrocarbons (PAHs) were detected in numerous fresh fracture surfaces (McKay *et al.*, 1996), which also included the previously mentioned carbonate globules. It was stated that the PAHs had a martian origin (Clemett *et al.*, 1998), although the meteorite was encased in Antarctic ice for 13,000 years and lay exposed on the surface for ∼500 years (McKay *et al.*, 1996). However, the presence of a filamentous organism observed on a fracture just beneath the fusion crust is proof of some terrestrial biogenic activity subsequent to ALH84001's fall to Earth (Steele *et al.*, 2000). Also, a ToF-SIMS analysis of ALH84001 specimens (Stephan *et al.*, 2003) revealed that the meteorite had been exposed to terrestrial contamination.
  Finally, a recent survey of the association of abiotic macromolecular carbon with magmatic minerals on several martian meteorites (ranging in age from 4.2 Ga to 190 Ma) indicates that martian magmas favor the precipitation of (abiotic) reduced carbon species during crystallization (Steele *et al.*, 2012). We, therefore, consider that the claim that ALH84001 PAHs may result from the action of past martian life is not sufficiently substantiated by the data.
  Associated with the carbonate globules' rims are also tiny crystals of magnetite (Fe_3_O_4_) and pyrrhotite (FeS). The magnetite crystals are 10–100 nm in size and are characterized by a particular prismatic crystallographic structure and very high chemical purity reported to be found only in biogenically formed magnetites (*i.e.*, in magnetosome chains, prokaryotic organelles acting like a compass needle to orient magnetotactic bacteria in geomagnetic fields) (Thomas-Keprta *et al.*, 2000, 2002). However, other works disputed this assertion on the basis that detailed morphologies of magnetite nanocrystals from three strains of magnetotactic bacteria were shown to differ from one another and none uniquely matched those in ALH84001 (Buseck *et al.*, 2001). Another study performed on ALH84001 material (Barber and Scott, 2002) concluded that the magnetite grains are abiogenic and formed by shock decomposition of carbonates in the meteorite. This explanation seems to be supported by shock recovery experiments carried out in the laboratory (Bell, 2007). In a later review article, Thomas-Keprta *et al.* (2009) argued that the chemical purity of the ALH84001 magnetite is not consistent with formation by thermal decomposition of the host carbonate and must have been added from an outside source, a scenario that does not exclude the possibility of a biogenic origin. It is our opinion that magnetite crystals (as observed on ALH84001) and, by extension, other tiny potential biominerals do not constitute a robust biosignature (especially for landed space missions) and have, therefore, not been included in our Figure 3 model. The score for chemical biosignatures is 0.
- *Discussion:* It is clear that the ExoMars rover payload would not be able to make any biosignature claims on a “difficult” sample like ALH84001. This is not surprising. It is like picking up a rock from a drawer in geology class that is completely removed from its context. The meteorite includes but a minimum of information regarding the regional environment and diagenetic history. It does not possess clear features combining morphological clues (candidate biofilms or fossilized microorganisms) with strong organic chemical signatures. The Kitty's Gap and Josefsdal Chert samples, however, are sedimentary rocks that formed in a better-understood setting. They were carefully selected from among many others based on their likely potential for preserving traces of life. We only have included ALH84001 here to discuss the applicability of a class of “more tenuous” possible biosignatures to space exploration.

#### 4.5.4. Yellowknife Bay, Mars

We next derive a score for Curiosity's analysis of samples obtained from two shallow (∼5 cm) drill holes, designated John Klein (J.K.) and Cumberland (C.B.), drilled into the lowermost stratigraphic unit, the Sheepbed member in the Yellowknife Bay formation, Gale Crater (Grotzinger *et al.*, 2014, 2015; Ming *et al.*, 2014; Bridges *et al.*, 2015; Freissinet *et al.*, 2015).

- *Geological context:* The two samples were interpreted to be mudstone formed in an ancient lacustrine environment; they contained ∼20wt % smectite clay (Bristow *et al.*, 2015). Terrestrial phyllosilicates like smectite can help to protect organic compounds when rapidly deposited under reducing chemical conditions. We grant 20 points for establishing the habitable nature of the water setting, both morphologically and through mineralogical analysis. It is not clear, however, that this was a widespread or very long-lived aqueous environment. Grotzinger *et al.* (2014) remarked that the wet period could have lasted anywhere between a few hundred years to tens of thousands of years. During this time, the paleo-lake environment could have supported the metabolism of modern-day terrestrial microbial life.
- *Morphological biosignatures:* No compelling macroscopic signs of ancient life were detected at the sites: no regular structures and no candidate biomediated layers. Numerous concretions were observed—potentially interesting targets (Stack *et al.*, 2014)—but could not be analyzed to evaluate the possible presence of organic biosignatures. Zero points.
- *Chemical biosignatures:* Pyrolysis (Pyr) of the J.K. and C.B. samples in the SAM (sample analysis at Mars) instrument led to the low temperature (125–350°C) release of chloromethane, C_2_–C_4_ dichloroalkanes, and chlorobenzene. This was the result of the thermal degradation of one or more oxychlorine compounds, such as perchlorate, that chlorinated organic species present in the sample. Freissinet *et al.* (2015) were able to separate the signal attributable to indigenous martian organics from a background signal caused by a residual derivatization agent (Glavin *et al.*, 2013). The authors concluded that the C.B. sample yielded 150–300 ppbw chlorobenzene and up to 70 ppbw C_2_–C_4_ compounds, released by Pyr of a previous organic precursor.
  The exogenous delivery of meteoritic organics (abiotic) to the martian surface has been estimated at ∼10^5^ kg C/year, mostly in the form of PAHs and kerogen that may undergo successive oxidation reactions. Therefore, a meteoritic source could have contributed the organic precursors needed for producing the detected chlorobenzene and dichloroalkanes (Freissinet *et al.*, 2015). However, the analysis of the Yellowknife Bay samples failed to detect any of the biosignatures shown in our Figure 3. Therefore, zero points.
- *Discussion:* A score of 20 means that, at the moment, we possess no indication to suggest that this area hosted life in the past.
  Likewise, we can neither confirm nor disprove the hypothesis by Noffke (2015) that morphological features observed elsewhere in Yellowknife Bay, in the sandstone beds of the Gillespie Lake member, could record the interaction of microbial mats with sediments. As suggested by the author, other supporting evidence, particularly chemical information, would be needed to further substantiate this possibility.

## 5. The Martian Environment and the Need for Subsurface Exploration

### 5.1. Results from previous missions

Based on what we knew about planetary evolution in the 1970s, many scientists regarded as plausible the presence of simple microorganisms on other planets. The 1976 Viking landers can be considered the first missions with a serious chance of discovering signs of life on Mars. That the landers did not provide conclusive evidence was not because of a lack of careful preparation. In fact, these missions were remarkable in many ways, particularly taking into account the technologies available. If anything, the Viking results were a consequence of the manner in which the life question was posed, seeking to elicit signs of microbial activity from potential extant ecosystems within the Mars samples analyzed (Klein *et al.*, 1976).

The twin Viking landers conducted the first *in situ* measurements on the martian surface. Their biology package contained three experiments, all looking for signs of metabolism in soil samples (Klein *et al.*, 1976). One of them, the Labeled-Release Experiment, produced very provocative results (Levin and Straat, 2016). If other information had not been also obtained, these data would have been interpreted as proof of biological activity. However, theoretical modeling of the martian atmosphere and regolith chemistry hinted at the existence of powerful oxidants that could, more or less, account for the results of the three biology package experiments (Klein, 1999). The biggest blow was the failure of the gas chromatograph mass spectrometer (GCMS) to acquire evidence of organic molecules at the parts-per-billion level. With few exceptions, the majority of the scientific community concluded that the Viking findings did not demonstrate the presence of extant life (Klein, 1998, 1999). At the time (Quinn *et al.*, 2007), numerous attempts were made in the laboratory to simulate the reactions observed by the Viking biological package. Although some reproduced certain aspects of the data, none succeeded entirely. The Viking program increased very significantly our knowledge of Mars; however, failure to detect organic molecules was considered a significant setback. As a consequence, our neighbor planet lost much of its allure. A multiyear gap in Mars surface exploration ensued.

The very successful 1996 Mars Global Surveyor and 2003 Mars Exploration Rovers (MER), which were conceived as robotic geologists, have demonstrated the past existence of wet environments (Malin and Edgett, 2000; Squyres *et al.*, 2004a, 2004b, 2012). But perhaps it has been Mars Express 2003 and Mars Reconnaissance Orbiter 2005 that have most drawn our attention to ancient Mars, revealing many instances of finely layered deposits containing phyllosilicate minerals that could only have formed in the presence of liquid water, which reinforced the hypothesis that early Mars was wetter than today (Poulet *et al.*, 2005; Bibring *et al.*, 2006; Loizeau *et al.*, 2010, 2012; Ehlmann *et al.*, 2011; Bishop *et al.*, 2013; Michalski *et al.*, 2013b).

The next incremental step in our chemical understanding of the martian surface was entirely unexpected. It came as a result of measurements conducted by the 2007 Phoenix lander in the northern subpolar plains. Phoenix included, for the first time, a wet chemistry analysis instrument that detected the presence of the perchlorate (ClO_4_^–^) anion in soil samples collected by the robotic arm (Hecht *et al.*, 2009; Kounaves *et al.*, 2010a, 2014). Perchlorates have interesting properties. For example, ammonium perchlorate is regularly used as a powerful rocket fuel oxidizer. Its salts are chemically inert at room temperature, but when heated beyond a few hundred degrees, the four oxygen atoms are released and become very reactive oxidation vectors. It did not take long for investigators to recall that Viking had relied on thermal volatilization (TV; in other words heat) to release organics from soil samples (Navarro-González *et al.*, 2010, 2011; Biemann and Bada, 2011; Navarro-González and McKay, 2011). If perchlorate had been present in the soil at the two Viking lander locations, perhaps heating could explain the negative organic carbon results obtained? In fact, some simple chlorinated organic molecules (chloromethane and dichloromethane) had been detected by the Viking experiments (Biemann *et al.*, 1977), but these compounds were interpreted to have resulted from a reaction between adsorbed residual methanol (a cleaning agent used to prepare the spacecraft) and HCl. Today, the general suspicion is that they were the outcome of heat-activated perchlorate dissociation and reaction with indigenous organic compounds (Steininger *et al.*, 2012; Glavin *et al.*, 2013; Quinn *et al.*, 2013; Sephton *et al.*, 2014; Goetz *et al.*, 2016; Lasne *et al.*, 2016).

On Earth, naturally occurring perchlorate-rich deposits are not that usual. They can be found in a few extremely dry environments, such as the Atacama Desert, in northern Chile (Catling *et al.*, 2010), and the Antarctic Dry Valleys (Kounaves *et al.*, 2010b). Typically a precursor, chlorine-containing volatile (*e.g.*, produced by nearby volcanism) plus a modicum of UV-photochemistry are required ingredients for their formation. However, recent studies show that purely gas phase atmospheric production is insufficient, by many orders of magnitude, to account for the perchlorate concentrations measured on Mars (Smith *et al.*, 2014; Carrier and Kounaves, 2015). Instead, the authors suggest that yet-to-be-identified, heterogeneous (*i.e.*, gas–solid surface) reactions occurring at UV-exposed, chloride-bearing mineral phases may be responsible. Perchlorate production on Mars may be happening at the surface, but could perhaps also involve reactions on lifted grains during dust storms, in a manner similar to that proposed by Atreya *et al.* (2006).

What can we extrapolate from this? Is perchlorate just a modern day phenomenon or has it always been a martian soil constituent? Is it to be found close to the surface only or does it run deep? Two lines of evidence inform our answer to these questions. The first is that we know Mars' atmosphere thinned much more rapidly than Earth's. The levels of UV light necessary to drive the formation of perchlorate precursors were reached on Mars billions of years ago, when volcanism was still active (Catling *et al.*, 2010; Carrier and Kounaves, 2015). This means that perchlorate, and any ionizing radiation-derived products (Quinn *et al.*, 2013), should be common also in very old deposits (certainly in Hesperian and Amazonian deposits), but perhaps less so in really ancient rocks formed when the atmosphere was denser (*i.e.*, the early Noachian). What about its distribution? We know from Earth that, once it has reached the soil, perchlorate can be very effectively dissolved and mobilized by water (Kalkhoff *et al.*, 2010; Cull *et al.*, 2014). It is, therefore, possible that sedimentary materials deposited under aqueous conditions (*i.e.*, mostly during the Noachian) may include less (or perhaps no) perchlorate. In contrast, salt-rich deposits resulting from ponding and subsequent evaporation may exhibit relatively high perchlorate concentrations. In other words, depending on the action of water as a transport *versus* concentration agent, we may observe variability in the distribution (and abundance) of perchlorate in ancient deposits. With no liquid water to wash it away, we can expect perchlorate to be mixed into any soil or rock formed after Mars became dry.

The second line of evidence comes from the findings of MSL's SAM experiment. The team detected oxygen (O_2_) released by the thermal decomposition of oxychlorine species (*i.e.*, perchlorates and/or chlorates (Archer *et al.*, 2016)), as well as chlorine-bearing hydrocarbons attributable to the reaction of oxychlorine species with organic compounds, when they analyzed modern sand deposits as well as when they drilled into much older rocks (Glavin *et al.*, 2013; Freissinet *et al.*, 2015). The inferred presence of perchlorate in the two different types of material (granular, recently transported and consolidated, ancient) cannot be explained by cross-contamination between samples. The ExoMars biosignature identification strategy needs to work also when the material to be analyzed contains perchlorate. We will see that this is indeed the case.

### 5.2. Degradation of organic matter

Effective chemical identification of biosignatures requires access to well-preserved organic molecules. Because the martian atmosphere is more tenuous than Earth's, three important physical agents reach the surface of Mars with adverse effects for the long-term preservation of biomarkers: (1) The UV radiation dose is higher than on our planet and will quickly damage exposed organisms or biomolecules. (2) UV-induced photochemistry is responsible for the production of reactive oxidant species that, when activated, can also destroy chemical biosignatures. The diffusion of oxidants into the subsurface is not well characterized and constitutes an important measurement that the mission must perform. Finally, (3) ionizing radiation penetrates into the uppermost meters of the planet's subsurface. This causes a slow degradation process that, operating over many millions of years, can alter organic molecules beyond the detection sensitivity of analytical instruments. Radiation effects are depth dependent: the material closer to the surface is exposed to higher doses than that buried deeper.

### 5.3. Access to molecular biosignatures

The molecular record of ancient martian life, if it ever existed, is likely to have escaped radiation and chemical damage only if trapped in the subsurface for long periods. Studies suggest that a subsurface penetration in the range of 2 m is necessary to recover well-preserved organics from the very early history of Mars (Kminek and Bada, 2006), assuming there has been some help from additional, recently eroded overburden (Dartnell *et al.*, 2007, 2012; Parnell *et al.*, 2007; Pavlov *et al.*, 2012). It is also essential to avoid loose dust deposits distributed by aeolian transport. In the course of being driven by the wind, this material has been processed by UV radiation, ionizing radiation, and potential oxidants in the atmosphere and on the surface of Mars. Any organic biosignatures would be highly degraded in these samples. For all the mentioned reasons, it was decided that the ExoMars rover must be able to penetrate and obtain samples from well-consolidated (*i.e.*, hard) formations, such as sedimentary rocks and evaporitic deposits, at various depths from 0 down to 2 m.

Having established that access to well-preserved subsurface deposits has a high scientific priority, why a deep drill? A large drill is expensive in terms of mission resources; it is also difficult to build and qualify for flight. Perhaps the team could have opted for a simpler solution: to include a mini corer having a shallower reach (*e.g.*, 5–10 cm) and rely on nature to have done the excavating through aeolian erosion and/or meteoritic impacts.

For a mission like ExoMars, access to the appropriate science target is the first factor to consider. The major difficulty with the investigation of biogenic material lies not in the recognition of fossil biosignatures, but in the ability to obtain the correct sample to study. As justified previously, it is water-lain sedimentary deposits from Mars' very early history that we are interested in. But not any old, wet location is suitable. We require ancient sites that have been uncovered by the action of wind only recently for molecular biosignature preservation against the ravages of long-term ionizing radiation. In the absence of a deep drill, the rover would need to drive close to a receding scarp to gain access to shallow material having experienced a lower radiation dose (Farley *et al.*, 2014). Not only samples of the right age, the right aqueous environment, the right deposit, and with the right exhumation history, but also from the foot of a scarp? How likely would that be? The ExoMars science team realized early on that having a subsurface drill greatly increases the probability to collect well-preserved material for analysis. It also provides the added bonus of being able to study how the geochemical environment changes with depth.

## 6. The ExoMars Rover and Its Pasteur Payload

### 6.1. From panoramic to molecular scale through nested investigations

The mission strategy to achieve the ExoMars rover's scientific objectives is as follows.

- To land on an ancient location possessing high exobiological interest for past life signatures, that is, access the appropriate geological environment.
- To collect well-preserved samples (free from radiation and oxidation damage) at different sites using a rover equipped with a drill capable of reaching well into the ground and surface rocks.
- To conduct an integral set of measurements at multiple scales to achieve a coherent understanding of the geological context and, thus, inform the search for biosignatures. Beginning with a panoramic assessment of the geological environment, the rover must progress to smaller scale investigations of surface rock textures and culminate with the collection of well-selected samples to be studied in its analytical laboratory.

**FIG. 4.**
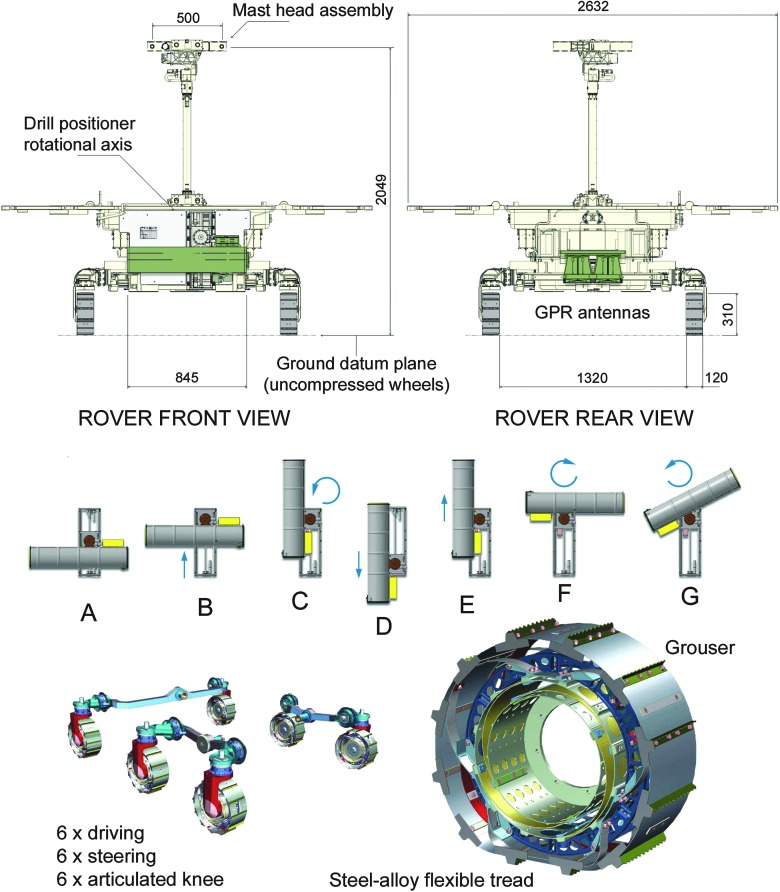
(Top) Front and rear views of the ExoMars rover with general dimensions (in mm). (Middle) The drill can acquire samples at depths ranging between 0 and 2 m. The drill box lies horizontally across the rover's front face when traveling **(A)**. It is raised **(B)**, rotated counterclockwise **(C)**, and lowered vertically to commence drilling operations **(D)**. Once a sample has been acquired, the drill is elevated **(E)**, turned clockwise **(F)**, and further inclined to deliver the sample **(G)**. The inlet port to the analytical laboratory can be seen on the rover's front, above the drill box, to the left. (Bottom) The rover's locomotion configuration is based on a triple-bogie concept and has flexible wheels to improve tractive performance.

The ExoMars rover will have a nominal lifetime of 218 sols (∼7 Earth months). During this period, it will ensure a regional mobility of several kilometers relying on solar array electrical power. Figure 4 presents front (top left) and rear views (top right) of the rover with some general size information. Its mass is ∼310 kg, with an instrument payload of 26 kg (excluding payload servicing equipment such as the drill and sample processing mechanisms).

The rover's kinematic configuration is based on a six-wheel, triple-bogie concept (Fig. 4 bottom left) with locomotion formula 6 × 6 × 6 + 6, denoting six supporting wheels, six driven wheels, and six steered wheels, plus six articulated (deployment) knee drives. This system enables the rover to passively adapt to rough terrains, providing inherent platform stability without the need for a central differential. The rover can perform drive and turn-on-spot maneuvers, double-Ackermann steering, and diagonal crabbing motions; the latter can be very useful for moving sideways across an outcrop for imaging.

Lander accommodation constraints have imposed the use of relatively small wheels (28.5 cm diameter without grousers, 12.0 cm width). To reduce the traction performance disadvantages of small wheels, flexible wheels have been adopted (Fig. 4 bottom right) (Favaedi *et al.*, 2011); their high deformation enlarges the size of the wheel–soil contact patch, reduces ground pressure (to ∼10 kPa average), and offers a substantial impact load absorption capability (Poulakis *et al.*, 2016). For comparison, the average wheel ground pressure of the MER (25 cm wheel diameter without grousers, 16 cm width) and MSL (48 cm wheel diameter without grousers, 40 cm width) rovers is 5.75 kPa (Heverly *et al.*, 2013). So the ExoMars rover exceeds the wheel ground pressure prescription of NASA rovers. This is a concern because, even with less wheel ground pressure, Opportunity experienced serious difficulties with unconsolidated terrain at Purgatory Ripple (Maimone *et al.*, 2007), and the same happened to MSL when attempting to traverse wind-blown, megaripple deposits (Arvidson *et al.*, 2016). To mitigate this risk, the ExoMars team is investigating the possibility to (re)enable wheel walking (Patel *et al.*, 2010), a coordinated rototranslational wheel gait that our tests have demonstrated can improve dynamic stability during rover egress, provide better traction for negotiating loose soils (in case the rover experiences excessive wheel slippage or gets stuck during normal driving), and increase slope gradeability (Azkarate *et al.*, 2015).

### 6.2. Pasteur payload instruments

**FIG. 5.**
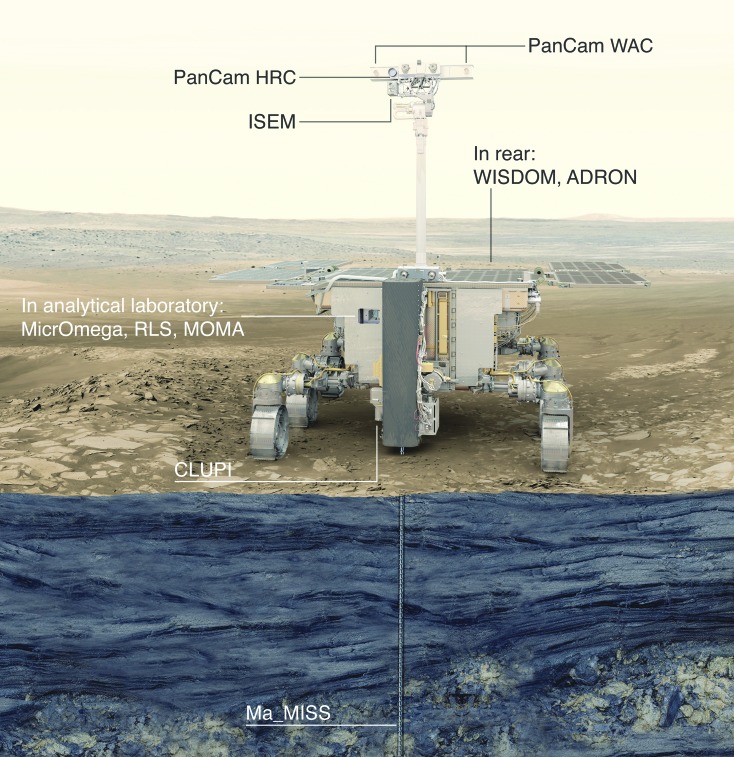
Sketch of ExoMars rover showing the location of the drill and the nine Pasteur payload instruments.

The rover's Pasteur payload will produce comprehensive sets of measurements capable of providing reliable evidence for, or against, the existence of a range of biosignatures at each search location. The Pasteur payload contains panoramic instruments [cameras, an infrared (IR) spectrometer, a ground-penetrating radar, and a neutron detector]; contact instruments for studying rocks and collected samples (a close-up imager and an IR spectrometer in the drill head); a subsurface drill capable of reaching a depth of 2 m and obtaining specimens from bedrock; a sample preparation and distribution system (SPDS); and the analytical laboratory, the latter including a visual + IR imaging spectrometer, a Raman spectrometer, and a laser desorption, TV GCMS (with the possibility to use three different derivatization agents)—see Figure 5 and Table 1.

**Table 1.** The ExoMars Rover's Pasteur Payload Can Perform a Detailed Mineralogical and Chemical Characterization of Surface and Subsurface Material Collected with the Drill

*Instrument*	*Scientific rationale*
Panoramic instruments	To characterize the rover's geological context, both at the surface and the subsurface. Typical scales span from panoramic (100 m) to 1 m, with a spatial resolution in the order of 1 cm for close targets.
Panoramic camera system	PanCam: Two wide-angle stereo cameras and one high-resolution camera to investigate the rover's environment and landing site geology. Also very important for target selection and for rock textural studies.
IR spectrometer	ISEM: For bulk mineralogy characterization, remote identification of water-related minerals, and for aiding PanCam with target selection.
GPR	WISDOM: To establish subsurface stratigraphy down to 3 m depth and help plan the drilling strategy.
Neutron detector	ADRON: To determine the level of subsurface hydration and the possible presence of an ice fraction to 1 m depth.
Contact instruments	To investigate outcrops, rocks, and soils. Among the scientific interests at this scale are macroscopic textures, structure, and layering. This information will be fundamental to understand the local depositional environment and to search for morphological biosignatures on rocks.
Close-up imager	CLUPI: To study rock targets at close range (50 cm) with sub-millimeter resolution. This instrument will also investigate the fines produced during drilling operations, and image samples collected by the drill. The close-up imager has variable focusing and can obtain high-resolution images also at longer distances. Certain morphological biosignatures, such as biolamination, if present, can be identified by CLUPI.
IR spectrometer in drill	Ma_MISS: For conducting mineralogical studies in the drill borehole's walls.
Support subsystems	These essential devices are devoted to the acquisition and preparation of samples for detailed studies in the analytical laboratory. The mission's ability to break new scientific ground, particularly for “signs of life” investigations, depends on these two subsystems.
Subsurface drill	Capable of obtaining samples from 0 to 2 m depth, where organic molecules can be well preserved from radiation damage. Includes a blank sample, temperature sensors, and an IR spectrometer (Ma_MISS).
Sample preparation and distribution system	Receives a sample from the drill system, produces particulate material preserving the organic and water fractions, and presents it to all analytical laboratory instruments. Includes a dispenser with additional blank samples.
Analytical laboratory	To perform a detailed, coordinated analysis of each collected sample. After sample crushing, the initial step is a visual and spectroscopic investigation. Thereafter follows a first search for organic molecules. In case interesting results are found, the instruments are able to perform more in-depth analyses.
VIS + IR imaging spectrometer	MicrOmega: Will examine the crushed sample material to characterize structure and composition at grain-size level. These measurements will be used to help point the laser-based instruments (RLS and MOMA).
Raman laser spectrometer	RLS: To identify mineral phases at grain scale in the crushed sample material, determine their composition, and establish the presence of carbon (inorganic/organic).
Mars organic molecule analyzer	MOMA (LD + Der-TV GCMS): MOMA is the rover's largest instrument. Its goal is to conduct a broad-range, very-high sensitivity search for organic molecules in the collected sample. It incudes two different ways of extracting organics: (1) LD and (2) TV, with or without derivatization (Der) agents, followed by separation using four GC columns. The identification of the evolved organic molecules is achieved with an ion trap MS.

ADRON, active detector for gamma rays and neutrons; CLUPI, close-up imager; GC, gas chromatograph; GPR, ground-penetrating radar; IR, infrared; ISEM, infrared spectrometer for ExoMars; LD, laser desorption; Ma_MISS, Mars multispectral imager for subsurface studies; MOMA, Mars organic molecule analyzer; MS, mass spectrometer; RLS, Raman laser spectrometer; SPDS, sample preparation and distribution system; TV, thermal volatilization; WISDOM, water, ice, and subsurface deposit observations on Mars.

If any bioorganic compounds are detected on Mars, it will be important to show that they were not brought from Earth. Great care is being devoted during the assembly, testing, and integration of instruments and rover components. Strict organic cleanliness requirements apply to all parts that come into contact with the sample and to the rover assembly process. Once completed, the analytical laboratory drawer (ALD) will be sealed and kept at positive pressure throughout transport, final integration, launch, cruise, and landing on Mars. The ExoMars rover will carry a blank in each drill tip (nominal and backup) to reliably demonstrate that the entire sample chain from acquisition through handling, processing, and analysis is free from contaminants. An additional six, individually encapsulated blanks will be stored in a dedicated dispenser. When deemed necessary, they can be used to evaluate the organic cleanliness of the sample handling and analysis chain. Upon landing, one of the first science actions will be for the drill to pass a blank sample to the analytical laboratory. After performing a full investigation, the results should indicate “no life” and “no organics.”

Hereafter, we provide a short summary of the Pasteur payload capabilities. Dedicated instrument articles can be found elsewhere in this issue.

#### 6.2.1. Panoramic camera system

Panoramic camera (PanCam) (Coates *et al.*, 2012, 2017 [this issue]; Cousins *et al.*, 2012; Yuen *et al.*, 2013) is designed to perform digital terrain mapping for the ExoMars rover mission. A powerful suite that consists of a fixed-focus, wide-angle, stereoscopic, color camera pair (WAC) complemented by a focusable, high-resolution, color camera (HRC), PanCam, will enable the science team to characterize the geological environment at the sites the rover will visit—from panoramic (tens of meters) to millimeter scale. It will be used to examine outcrops, rocks, and soils in detail, and to image samples collected by the drill before they are delivered to the analytical laboratory for analysis. PanCam will also be a valuable asset for conducting atmospheric studies.

*PanCam WAC:* 1024 × 1024-pixel, multispectral, stereoscopic images with 32.28° (horizontal/vertical) field of view (FOV).

*PanCam HRC:* 1024 × 1024-pixel, color, monoscopic images with “telephoto” 4.88° (horizontal/vertical) FOV.

#### 6.2.2. IR spectrometer

ISEM (Korablev *et al.*, 2015, 2017 [this issue]) is a pencil-beam IR spectrometer mounted on the rover mast that is coaligned with the PanCam high-resolution camera. ISEM will record IR spectra of solar light reflected off surface targets, such as rocks and soils, to determine their bulk mineralogical composition. ISEM will be a very useful tool to discriminate between various classes of minerals at a distance. This information can be employed to decide which target to approach for further studies. ISEM can also be used for atmospheric studies.

*ISEM:* 1.1–3.3-μm spectral range, 20 cm^–1^ spectral sampling, with 1° FOV.

#### 6.2.3. Shallow ground-penetrating radar

The WISDOM (water, ice, and subsurface deposit observations on Mars) radar (Ciarletti *et al.*, 2011, 2017 [this issue]) will characterize stratigraphy to a depth of 3–5 m with vertical resolution of the order of a few centimeters (depending on subsurface electromagnetic properties). WISDOM will allow the team to construct two- and three-dimensional subsurface maps to improve our understanding of the deposition environment. Most importantly, WISDOM will identify layering and help select interesting buried formations from which to collect samples for analysis. Targets of particular interest for the ExoMars mission are well-compacted, sedimentary deposits that could have been associated with past water-rich environments. This ability is fundamental to achieve the rover's scientific objectives, as deep subsurface drilling is a resource-demanding operation that can require several sols.

*WISDOM:* broad-band UHF GPR (0.5–3.0 GHz), step frequency, bistatic and polarimetric (XX-XY-YX-YY) measurements, penetration depth ∼3 m, vertical resolution of a few centimeters.

#### 6.2.4. Subsurface neutron detector

ADRON (active detector for gamma rays and neutrons) (Mitrofanov *et al.*, 2017 [this issue]) will count the number of thermal and epithermal neutrons scattered in the martian subsurface to determine hydrogen content (present as grain adsorbed water, water ice, or in hydrated minerals) in the top 1 m. This information will complement the subsurface characterization performed by WISDOM.

*ADRON:* detects neutrons in the broad range 0.01 eV to ∼100 keV.

#### 6.2.5. Close-up imager

Close-up imager (CLUPI) will obtain high-resolution, color images to study the depositional environment (Josset *et al.*, 2017 [this issue]). By observing textures in detail, CLUPI can recognize potential morphological biosignatures, such as biolamination, preserved on surface rocks. This is a key function that complements the possibilities of PanCam when observing close targets at high magnification. CLUPI will be accommodated on the drill box and have several viewing modes, allowing the study of outcrops, rocks, soils, the fines produced during drilling, and also imaging collected samples in high resolution before delivering them to the analytical laboratory.

*CLUPI:* 2652 × 1768-pixel, color, z-stacked images, 11.9° × 8.0° FOV, imaging resolution varies with distance to target, for example, it is 8 μm/pixel at 11.5 cm distance with view area 2.0 × 1.4 cm, 39 μm/pixel at 50 cm distance with view area 10 × 7 cm, and 79 μm/pixel at 100 cm distance with view area 21 × 14 cm.

#### 6.2.6. Drill IR spectrometer

Ma_MISS (Mars multispectral imager for subsurface studies) (De Angelis *et al.*, 2013; De Sanctis *et al.*, 2017 [this issue]) is a miniaturized IR spectrometer integrated in the drill tool for imaging the borehole wall as the drill is operated. Ma_MISS provides the capability to study stratigraphy and geochemistry *in situ*. This is important because deep samples may be altered after their extraction from their cold, subsurface conditions, typically of the order of −50°C at mid latitudes (Grott *et al*., 2007). The analysis of unexposed material by Ma_MISS, coupled with other data obtained with spectrometers located inside the rover, will be crucial for the unambiguous interpretation of rock formation conditions.

*Ma_MISS:* 0.4–2.2 μm spectral range, 20 nm spectral sampling, with spatial resolution of 120 μm (corresponding to one rotational step of the drill tool).

#### 6.2.7. Subsurface drill

ExoMars employs a rotary drill (with no percussion capability) to acquire (∼3-cm-long by 1-cm-diameter) samples (solid, fragments, or powder) at depths ranging between 0 and 2 m. The drill box lies horizontally across the rover's front face when traveling (Fig. 4A); it assumes a vertical stance for drilling (Fig. 4D) and is raised and rotated for delivering a sample to the analytical laboratory's inlet port (Fig. 4G). The drill box's dexterity is also used for orienting CLUPI observations (CLUPI is not shown in sketches A to G).

The drill is composed of the following elements: (1) a drill tool ∼70 cm long, equipped with the sample acquisition device (including a shutter, movable piston, position, and temperature sensors) and the Ma_MISS front elements (sapphire window, IR lamp, reflector, and optical fiber); (2) a set of three extension rods, 50 cm each, to achieve the required penetration depth, they contain optical and electrical contacts for the transmission of Ma_MISS signals to the spectrometer in the upper part of the drill unit; (3) a backup drill tool without spectrometer; and (4) the rotation–translation group, comprising sliding carriage motors, guides, and sensors.

Preserving the sample's organic and volatile content is of paramount scientific importance. The drill has thermocouples close to the tip to monitor temperature variations in the sample collection region. We have conducted numerous tests in Mars chambers using different, geologically representative, simulated stratigraphic columns, including ice lenses varying from 0% to 35% water content. Temperature increases because of drilling are ephemeral and modest, in the order of 20°C, when we proceed in a continuous manner, and can be reduced to ≤5°C if we implement a variable cutting law (“cut a little, wait a little” to allow dissipating thermal energy) just before collecting the sample; however, this means more time. The low atmospheric pressure on Mars leads to the rapid sublimation of any ice particles directly in contact with the drill tip, resulting in an upward traveling gas jet that can be very helpful for evacuating drill fines from the borehole. Considering the typical temperature of subsurface materials on Mars (which at midlatitudes can experience oscillations between −30 and −80°C at 0.5 m depth and have an average value of about −50°C deeper), we can adapt our strategy to ensure that samples remain sufficiently cold throughout the drilling process.

#### 6.2.8. Sample preparation and distribution system

**FIG. 6.**
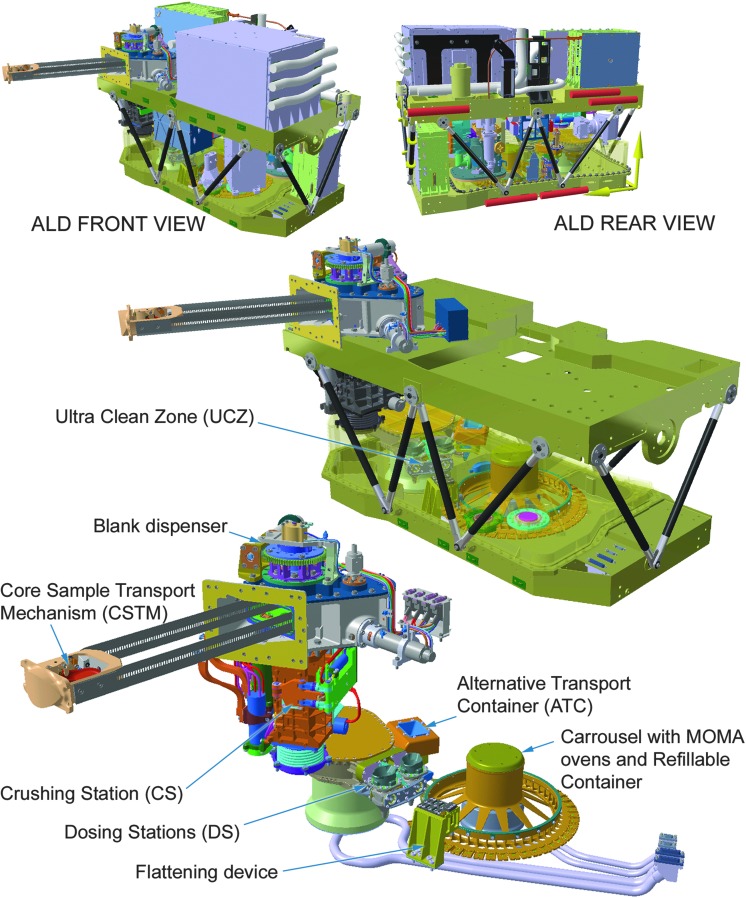
(Top) Front and rear depictions of the ExoMars rover ALD housing MicrOmega, RLS, MOMA, and the SPDS. (Middle) The UCZ envelops the entire sample-handling path and is sealed at positive pressure until open on Mars. (Bottom) SPDS mechanisms: The sample is deposited in the CSTM and, after being imaged with CLUPI and PanCam, is retracted into the ALD. The rock CS crushes the sample and discharges the resulting particulate matter into a DS. The DS pays out the necessary amount of sample material onto the refillable container or into a MOMA oven, as necessary. ALD, analytical laboratory drawer; CLUPI, close-up imager; CS, crushing station; CSTM, core sample transport mechanism; DS, dosing station; MOMA, Mars organic molecule analyzer; SPDS, sample preparation and distribution system; UCZ, ultra clean zone.

The entire ALD sample path is enclosed in a so-called ultra clean zone (UCZ), which is shown as a transparent volume in Figure 6 (middle). The SPDS groups the ensemble of ALD mechanisms used for manipulating sample material (Fig. 6 bottom). The SPDS receives a sample from the drill by extending its core sample transport mechanism (CSTM), a sort of *hand* that comes out through a door in the rover's front panel (shown in Fig. 5). Once deposited in the CSTM, typically at the end of a sol's work, PanCam HRC and CLUPI can image the sample during a narrow time window of a few minutes; this duration is based on the results of a sample contamination analysis from possible external rover system sources.

After the imaging exercise has been completed, the CSTM retracts, moving the sample into the analytical laboratory. A rock crusher is used to produce particulate matter having a more or less Gaussian size distribution ranging from a few to ∼500 μm, with 250 μm as the median value. This is done very early in the morning, when the temperature in the ALD is at its lowest, to preserve as much as possible the organic and volatile fractions in the sample. The temperature of the crushing station (CS) is monitored before and throughout the crushing process. The SPDS includes a blank dispenser with the capability to provide individual blank samples to the rock crusher. The pulverized sample material drops into one of two, redundant dosing stations (DSs). Their function is to distribute the right amount of sample either to a refillable container—a flat tray where mineral grains can be observed by ALD instruments—or into individual, single-use ovens. A rotating carrousel accommodates the refillable container and ovens under the DS. Both DSs are piezovibrated to improve the flow of granular material. The refillable container is further served by two other mechanisms: the first flattens the crushed sample material at the correct height to present it to the ALD instruments and the second is utilized to empty the refillable container so that it can be used again.

A number of emergency devices have been implemented to deal with potential off-nominal situations. To prevent the CS from becoming blocked, a spring-actuated hammer can apply a strong shock to the fixed jaw, where material may stick. In case of jamming, a special actuator can open the CS jaws to evacuate the entire sample. If both DSs were to fail, they can be bypassed. An alternative transport container allows dropping the entire crushed sample material at once, without control for the quantity provided, either onto the refillable container or into an oven.

#### 6.2.9. MicrOmega

Micro observatoire pour la mineralogie, l'eau, les glaces et l'activité (MicrOmega) (Pilorget and Bibring, 2013; Bibring *et al.*, 2017 [this issue]) will be the first instrument to image the crushed sample material. MicrOmega is a very-near IR hyperspectral camera that will study mineral grain assemblages in detail to try to unravel their geological origin, structure, and composition. Its FOV covers a sample area of 5 × 5 mm^2^. These data will be vital for interpreting past and present geological processes and environments on Mars. The rover computer can analyze a MicrOmega hyperspectral cube's absorption bands at each pixel to identify particularly interesting minerals and assign them as objectives for Raman and MOMA-laser desorption mass spectrometry (LDMS) observations.

*MicrOmega:* 250 × 256-pixel × 320-spectral step VIS + IR image cubes, 0.95–3.65 μm spectral range, 20 cm^–1^ spectral sampling, with imaging resolution of 20 μm/pixel.

#### 6.2.10. Raman laser spectrometer

Raman laser spectrometer (RLS) (Edwards *et al.*, 2013; Foucher *et al.*, 2013; Lopez-Reyes, 2015; Rull *et al.*, 2017 [this issue]) provides geological and mineralogical information on igneous, metamorphic, and sedimentary processes, especially regarding water-related interactions (chemical weathering, chemical precipitation from brines, etc.). In addition, it also permits the detection of a wide variety of organic functional groups. Raman can contribute to the tactical aspects of exploration by providing a quick assessment of organic content before the analysis with MOMA.

*RLS:* continuous excitation, 532 nm (green laser) with a 50-μm-size spot on the target, covering an ∼150–3800-cm^–1^ spectral shift with Raman resolution ∼6 cm^–1^ in the fingerprint spectral region <2000 cm^–1^ and with slightly degraded spectral resolution beyond this value.

#### 6.2.11. Mars organic molecule analyzer

MOMA is the largest instrument in the rover, and the one that directly targets chemical biosignatures. MOMA is able to identify a broad range of organic molecules with high analytical specificity, even if present at very low concentrations (Arevalo *et al.*, 2015; Goetz *et al.*, 2016; Goesmann *et al.*, 2017 [this issue]).

MOMA has two basic operational modes: LDMS, to study large macromolecules and inorganic minerals (Busch, 1995; Bounichou, 2010), and GCMS, for the analysis of volatile organic molecules. In MOMA-LDMS, crushed drill sample material is deposited in a refillable container. A high-power, pulsed UV laser fires on the sample and the resulting molecular ions are guided into the mass spectrometer for analysis. In MOMA-GCMS, sample powder is deposited into one of 32 single-use ovens. The oven is sealed and heated up stepwise to a high temperature (for some ovens, in the presence of a derivatization agent). The ensuing gases are separated by gas chromatography and delivered to the shared mass spectrometer for analysis. This combined process of derivatization, chromatographic separation, and mass spectrometric identification is useful for small organic molecules, such as amino acids.

The MOMA instrument implements innovative techniques for the extraction and robotic characterization of organic molecules, including the derivatization of refractory molecules such as carboxylic acids and amino acids. For the elucidation of the chirality of martian analytes, the MOMA gas chromatograph employs one chiral stationary phase that is able to resolve and quantify enantiomers of many different families of organic molecules. Furthermore, the MOMA-LDMS mode of operation does not seem to be affected by the presence of perchlorate in the sample (Li *et al.*, 2015); the laser energy deposition pulse is too fast for perchlorates to dissociate and trigger oxidative reactions, but effective enough to desorb organic molecules.

An early MOMA-GCMS prototype was tested in the field during the AMASE11 field Campaign in Svalbard, Norway (Siljeström *et al.*, 2014).

*MOMA Pyr GCMS:* 20 pyrolysis ovens, each ∼150 mm^3^ sample volume heated to any desired temperature <800°C, four different GC columns (including one enantioselective). For volatile organics (*e.g.*, alkanes, amines, alcohols, and carboxylic acids), detection mass range of 50–500 Da, detection limit ≤ nmol analyte [signal-to-noise ratio (SNR ≥ 10)].

*MOMA derivatization (Der) GCMS and thermochemolysis (Pyr + Der) GCMS:* four ovens for each of three derivatization agents: (1) MTBSTFA/DMF (for carboxylic and amino acids, nucleobases, amines, and alcohols), (2) DMF-DMA (for amino acids, fatty acids, and primary amines with chiral centers; this agent preserves the asymmetric center C* so will be used together with the enantioselective GC column for chiral separation), and (3) TMAH (for lipids, fatty acids, and—when driven to higher temperatures, *e.g.*, 700°C—for more refractory compounds such as PAHs and kerogen); each ∼150 mm^3^ sample volume heated to some moderately high temperature from 100 to 300°C; detection mass range of 50–500 Da, detection limit ≤ nmol analyte (SNR ≥ 10).

*MOMA LDMS:* λ = 266 nm UV laser, ∼1.3 ns, ≤135 μJ pulses in bursts ≤ 100 Hz (average 2 Hz), with a spot size of 400 × 600 μm and a depth of 10 nm/shot. For nonvolatile organics (*e.g.*, macromolecular carbonaceous compounds, proteins, and inorganic species), detection mass range of 100–1000 Da, detection limit ≤ pmol/mm^2^ analyte (SNR ≥ 3).

### 6.3. The reference surface mission

The reference surface mission (RSM) defines a rover exploration scenario that has two *raisons d'être*. The first is scientific. The RSM specifies a logical sequence of science steps, proceeding from large- to small-scale studies, concluding with the collection and analysis of samples from both surface and subsurface targets. The RSM is ambitious, and thus affords sufficient rover resources and operational scope to carry out something completely different in case the landing site would not match our expectations. For instance, the rover could travel a distance far longer than originally planned to reach a suitable prime science location, at the expense, of course, of time for investigations. The second purpose of the RSM is formal. When ESA placed a contract with European industry to procure the ExoMars rover, it specified that it must be capable of executing the RSM within the nominal mission duration. The demonstration—through simulation and tests—that the rover can complete the RSM is an agreed requirement.

**FIG. 7.**
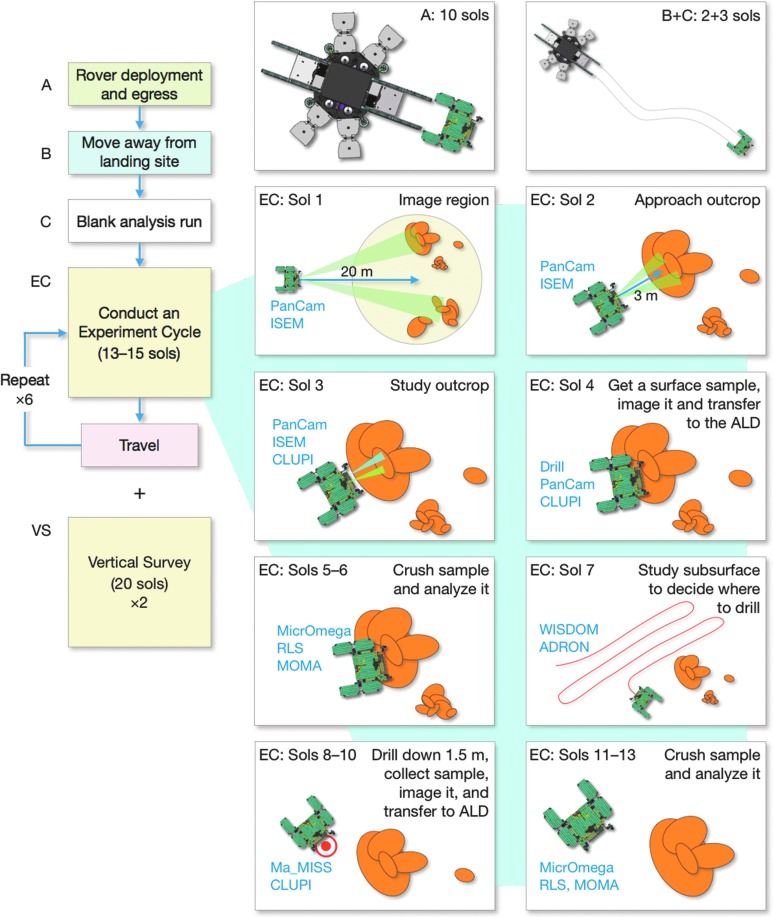
Major activities in the rover RSM include six ECs and two VSs. The VSs will be conducted at particularly interesting locations identified during the course of the mission. ECs, experiment cycles; RSM, reference surface mission; VSs, vertical surveys.

Figure 7 presents the various steps in the ExoMars rover RSM. The RSM begins with the rover deployment and egress sequence in either of two possible directions. Thereafter, a short functional commissioning phase is performed in the vicinity of the surface platform (Fig. 7A, duration 10 sols). The rover then moves (∼60 m) away from the descent engine blast contamination zone. Once sufficiently far, the drill and the ALD may be opened. The first ALD science operation is the drill blank analysis run to perform a full calibration and assess organic cleanliness. Only after this has been completed can the search for biosignatures begin (Fig. 7B + C, duration 5 sols).

The exploration part of the RSM includes six experiment cycles (ECs) and two vertical surveys (VSs). During the course of an EC, the rover exercises all Pasteur instruments and analyzes two samples, one surface and one subsurface—the latter specified to be obtained at 1.5 m depth. The distance between EC locations is arbitrarily assumed to increase in 100-m steps (100 m to the first spot, an additional 200 m to the second, and so on) for a total surface travel of ∼1.5 km. Twice during the nominal mission, most likely in case something particularly interesting is found, a VS can be executed. During a VS, samples are collected (and analyzed) at 0-, 50-, 100-, 150-, and 200-cm depths from the same place. The objective of a VS is to understand how organic compound preservation and overall geochemistry vary with depth.

At least for the first few months, rover operations will be conducted on “Mars time.” We assume two communication sessions per sol with Earth through TGO; this is the nominal condition. Typically, ground control instructs the rover what to do during a morning pass and the rover reports back the results of its travails in the evening pass. This means that all critical data required to define the next sol's activities must reach the Rover Operations and Control Center (ROCC) with the evening pass. However, since TGO's orbit is not Sun-synchronous, the local time of communications sessions drifts in a complex but (fortunately) predictable manner—they take place ∼30 min earlier each sol for three consecutive sols, then jump forward ∼2 hr to start the cycle again. Moreover, the duration and data volume capacity of TGO overflights are not constant because of the varying geometry between TGO and the landed asset. These two conditions introduce an additional constraint on strategic operations planning: it may not always be possible to complete the required tasks and provide the data “just in time” for the next TGO pass. Under such circumstances we would need to tailor rover activities to the available time (as part of the daily tactical planning exercise) or accept to skip a communications session.

Sol 1 of an EC considers that the rover is at ∼20 m from an interesting area. It gathers PanCam (panoramic and high-resolution) and ISEM data to identify potential targets of interest, which it relays to ROCC. On Sol 2, the rover approaches an Earth-designated outcrop to 3 m distance and obtains visual and spectral information. Along the way it uses WISDOM and ADRON to study the subsurface. On Sol 3, the rover conducts a high-resolution inspection with CLUPI of a portion of the outcrop to better understand the lithology and investigate interesting textures. On Sol 4, the rover collects a surface sample from the outcrop with the drill. CLUPI and PanCam HRC image the sample, which is then delivered to the ALD. Sol 5 starts with the crushing of the sample early in the morning when it is coldest. The grains are imaged with MicrOmega, which identifies mineral phases of interest and instructs the SPDS carrousel to move those parts of sample space under RLS for Raman spectroscopic studies—RLS can also perform automatic sequences—and MOMA for laser desorption investigations. This triad of observations (MicrOmega, RLS, and MOMA-LDMS) provides a first taste of a sample's mineral composition and potential organic content. Knowledge from all three ALD instruments' findings is needed for planning any additional work on the material. For Sol 6, we have assumed using RLS further and then MOMA-GCMS.

During Sol 7, the rover executes a subsurface scanning pattern with WISDOM and ADRON with the objective of selecting a suitable place for drilling deep. WISDOM is used to identify the distance (depth) to the target sedimentary layers and make sure there are no buried obstacles in the way. ADRON contributes valuable data about the level of hydration, which can inform the drilling and sample crushing strategy: do we need to worry about water content in the sample leading to possible particle cementation? The rover moves to the desired position and starts drilling: 50 cm on Sol 8, a further 50 cm on Sol 9, and the final 50 cm on Sol 10 to reach the 1.5 m depth assumed for this exercise. On each day, Ma_MISS studies the borehole material *in situ*, and CLUPI images the fines accumulating in the drill mound (the fines cannot be seen by PanCam while drilling). The subsurface sample is collected, imaged with CLUPI and PanCam HRC, and transferred to the ALD. Sol 11 is like Sol 5: MicrOmega, RLS, and MOMA-LDMS are utilized to get a first idea of what the sample holds. On Sol 12, RLS and MOMA-LDMS conduct a more in-depth analysis of the sample material. Finally, on Sol 13 a complete MOMA-GCMS analysis takes place. Thereafter, the rover moves somewhere else, assuming a rover traverse of ∼100 m per sol, to commence another EC.

The secret to successful deep drilling is to proceed slowly. The RSM assumes a conservative vertical progress of 50 cm per sol, mostly through loose regolith, to reach the target sedimentary deposit at a prescribed depth. What can actually be achieved on any given sol will depend on rover resources, the nature of the terrain being drilled, and the time available. Progress will be less when the terrain is harder and/or the drill goes deeper. We have demonstrated that the tool can sample formations of up to 150 MPa unconfined compressive strength (this covers most sedimentary rocks and weathered basalt, but not hard basalt or chert) and collect cores, fragments, or unconsolidated material (Magnani *et al.*, 2011).

The desired baseline approach for drill operations is to be able to “park” (a part of) the drill string in the subsurface overnight, minimizing “dead” string assembly/disassembly periods to afford Ma_MISS sufficient science time. Operationally, however, this will be achieved in a stepwise manner. In the beginning, the rover will disassemble and store all segments at the end of each sol. Thereafter, part of the tip will be left in the borehole to evaluate the torque necessary to get it to move again in the morning. As confidence builds up, progressively longer drill sections will be allowed in the borehole overnight.

Tests performed in a Mars atmospheric chamber during which the drill was exercised to its full penetration length through different Mars-representative stratigraphic sequences permeated with 10–35% water-content ice lenses, stopped, and left in the (simulated) subsurface overnight at −110°C, showed that it was possible to restart the drill in the morning and extract it safely out of the borehole. Nonetheless, if the drill were to get stuck, it is feasible to command its counter rotation to disengage the string at the last blocked element, recovering the top portion. Further drilling would need to be performed with the backup drill tool and any remaining extension rods. The positioning system is equipped with an emergency ejection unit to be used as a last resort in case the drill becomes permanently immobilized in the terrain. However, without the drill, it would no longer be possible to provide samples to the ALD.

Summarizing, the RSM provides a step-by-step model exploration scenario that indicates how the mission objectives could be fulfilled. Its scope secures a level of resources affording a good degree of operational flexibility. Nevertheless, the real mission is likely to be very different. The rover and instrument teams will adapt science operations as necessary to perform the best possible mission with the available resources.

## 7. A Suitable Landing Site

Barring the minor issues of landing and egressing safely, it is the scientific characteristics of the landing site region that will have the greatest effect on what the ExoMars rover will be able to discover. Attributes such as (1) age; (2) nature, duration, and connectivity of aqueous environments; (3) sediment deposition, burial, diagenesis, and (4) exhumation history are decisive for the successful (or otherwise) trapping and preservation of possible chemical biosignatures. Other aspects related to how we may gain access to good samples are also important. For example, how many prime targets can we identify from orbit? What is their relative spacing and distribution in the landing ellipse? Do obstacles exist for rover locomotion? How extensive?

During 2013, ESA and Roscosmos appointed a Landing Site Selection Working Group (LSSWG) for the second ExoMars mission. The LSSWG includes the necessary scientific and engineering expertise to evaluate the suitability of candidate landing sites to meet science, engineering, and planetary protection constraints (Vago *et al.*, 2015). Combining scientific and engineering competence in one body was considered paramount to the success of the landing site selection process. Two separate bodies, one scientific and another engineering, would have likely resulted in incompatible recommendations. In this manner, the successful combination of science interest and landing safety must be achieved within the LSSWG.

### 7.1. Scientific constraints

The ExoMars rover mission must target a geologically diverse, ancient site interpreted to posess strong potential for past habitability and for preserving physical and chemical biosignatures (as well as abiotic/prebiotic organics).

1. *Age:* The site must be older than 3.6 Ga, from Mars' early habitable period: pre- to late-Noachian (Phyllosian), possibly extending a bit into the Hesperian.
2. *Preservation:* Regarding the search for molecular biosignatures, the site must provide easy access to locations with reduced radiation accumulation in the subsurface. The presence of fine-grained sediments in units of recent exposure age would be very desirable (on Earth, organic molecules are better preserved in fine-grained sediments—which are more resistant to the penetration of biologically damaging agents, such as oxidants—than they are in porous, coarse materials). Young craters can provide the means to access deeper sediments, and studies on Earth suggest that fossil biomarkers can survive moderate impact heating (Parnell and Lindgren, 2006). In addition, impact-related hydrothermal fractures might have contributed to creating habitats for microbial life in the past. However, for landing safety reasons, it is better not to have many craters in the ellipse, so sites recently exposed by high erosion rates would be preferable.
3. *Aqueous history:* The site must show abundant morphological and mineralogical evidence for long-duration (preferred), or frequently reoccurring (acceptable), low-energy transport, aqueous activity. We seek a geological setting with a water-rich/hydrothermal history consistent with conditions favorable to life (*e.g.*, evidence of slow-circulating or ponded water).
4. *Outcrop distribution:* The site must include numerous sedimentary rock outcrops. The outcrops must be well distributed over the landing ellipse to ensure that the rover can get to some of them.
5. *Little dust and drift sand:* It is essential to avoid loose dust deposits and drift sand distributed by aeolian transport. Scientifically, there are two reasons for this: (1) Dust and mobile sand are not an interesting target for the rover. (2) The usefulness of the drill will be nullified if the landing site has a dust/sand layer thicker than the drill's maximum penetration depth. In addition, dunes constitute a serious risk for the rover's locomotion system.

### 7.2. Engineering constraints

Engineering constraints are criteria that, in case they are not satisfied, can result in a landing site being judged unfeasible for the mission and therefore rejected.

1. *Altitude:* The terrain elevation in the landing ellipse must be less than or equal to −2 km MOLA.
2. *Landing ellipse size:* Including margin to account for off-track radar operations (*i.e.*, while oscillating under the parachute), the initial landing ellipse for site selection has been assumed to be 104 × 19 km, although it may vary according to the selected site's location and other dynamical constraints on trajectory imposed by the launch period.
3. *Terrain relief:* Surface features and slopes are entry, descent, and landing performance drivers because they can impact radar measurements and affect the stability of the landing platform. They can also constitute trafficability obstacles for the rover.
4. *Rock distribution:* The landing platform is designed with a clearance between nozzles and terrain of 0.50 m as the legs touch down, and 0.35 m after deformation of the legs' shock absorbers. The site must have an areal fraction occupied by surface rocks (commonly referred to as rock abundance) ≤7%.
5. *Latitude:* The ExoMars rover can operate in the latitude range between 5 and 25°N.

These engineering constraints and others; including thermal inertia, aeolian deposits cover, radar reflectivity, and wind speed limits; are more precisely described in the work of Vago *et al.* (2015).

### 7.3. Planetary protection constraints

The ExoMars mission is not compatible with landing or operating in a Mars Special Region (Kminek and Rummel, 2015; Kminek *et al.*, 2016). For the mission to be able to access a location where Earth microorganisms could multiply, the complete lander plus rover combination would need to be sterilized to satisfy Category 4c bioburden levels (as was done for the Viking landers). This will not be the case. Instead, the ExoMars mission has been classified as Category 4b. It is a mission including analytical instruments that can detect signatures of extinct and extant life; hence, all parts of the spacecraft that can come into contact with samples (*i.e.*, the drill, the SPDS, and all mechanisms and volumes) have to be isolated, organically clean, and sterile throughout the mission to avoid potential false positive detections (as per Category 4b rules). The rest of the rover (and indeed the lander) will comply with Category 4a prescriptions, those used for the MSL and MER rovers. A Category 4b classification allows exploring for signs of (extinct) life outside Mars Special Regions. Since ExoMars focuses on the search for ancient life biosignatures and landing site selection is tailored accordingly, this is the right approach.

The work to ensure that a candidate landing site does not include surface features that must be treated as Special Region (evaluated on a case-by-case basis), or experience environmental conditions that would meet the threshold levels of the parameters defined for Special Regions, is based on a detailed analysis of orbital data, laboratory-based experiments, and modeling. A team appointed by the European Science Foundation will perform an independent review of the mission team's results.

### 7.4. Possible locations for landing

Two candidate landing sites have been identified: Oxia Planum and Mawrth Vallis (Bridges *et al.*, 2016) (Fig. 1F). Both will need to be verified in detail before the final landing location can be selected.

#### 7.4.1. Oxia Planum (18.159°N, 335.666°E; −3 km MOLA)

The Oxia Planum area is situated at the eastern margin of the Chryse basin, along the martian dichotomy border, and at the outlet of the Coogoon Valles system. The ellipse lies in the lower part of a wide basin where extensive exposures of Fe/Mg-phyllosilicates (>80% of the ellipse surface area) have been detected with both OMEGA and CRISM hyperspectral and multispectral data (Quantin *et al.*, 2016). Smectite clays (Fe–Mg-rich saponite) or smectite/mica (*e.g.*, vermiculite) are the dominant minerals within the ellipse. Hydrated silica, possibly opal, and Al-rich phyllosilicates may be present to the east of the ellipse (Carter *et al.*, 2016). The Fe/Mg-rich clay detections are associated with early/middle- to late-Noachian layered rocks (with layering thickness ranging from a few meters to <1 m for several tens of meters). They may represent the southwestern expansion (lowest member) of the Mawrth Vallis clay-rich deposits, pointing to a geographically extended aqueous alteration process.

The large Fe/Mg phyllosilicate-bearing unit overlaps the pre-existing topography, is cut by valleys and inverted channels, and is overlain by younger, presumably Hesperian, alluvial, and deltaic sediments to the east of the ellipse. A 10-km wide, 80-km long, low thermal inertia feature interpreted as a potential delta, and bearing hydrated silica signatures in its stratum, is observed at the outlet of Coogoon Valles. The putative delta waterline suggests the presence of a standing body of water after the formation of the clay-rich unit over the entire landing ellipse area (Quantin *et al.*, 2016). A 20-m-thick, dark, capping unit covers both the layered formation and the fluvial morphologies, and is interpreted to be Amazonian lava material. Crater counts yield ages of 4.0 Ga for the clay-rich unit and 2.6 Ga for the capping unit. The region has undergone extensive aeolian erosion, as attested by anomalies in crater density, forming geological windows to fresh exposures (<100 Ma) where material has been recently removed (Quantin *et al.*, 2016).

#### 7.4.2. Mawrth Vallis (22.160°N, 342.050°E; −2 km MOLA)

The Mawrth Vallis area is located at the boundary between the cratered Noachian terrains and the northern lowlands and represents one of the largest exposures of phyllosilicates detected on Mars (Poulet *et al.*, 2005; Bibring *et al.*, 2006; Loizeau *et al.*, 2007). The proposed ellipse lies in early/middle- to late-Noachian clay-rich terrains southwest of the Mawrth Vallis channel (Gross *et al.*, 2016).

The phyllosilicates are arranged in light-toned, finely layered deposits (∼1 m thickness) of unknown origin, but their extent—covering thousands of square kilometers—is suggestive of a large, stable aqueous system. Outcrops in Mawrth Vallis are compositionally diverse, with a >300 m thick sequence of various Al-phyllosilicates overlying Fe/Mg-smectites, including local outcrops of sulfates (alunite, jarosite, and bassanite) and hydrated silica (Poulet *et al.*, 2014).

These rocks show the highest degree of mineralogical diversity identified so far on Mars, which suggests a rich geological history that may have included multiple aqueous environments. The deposition and aqueous alteration of the smectites are ancient (dated at 4.0 Ga) and have most likely been followed by episodes of acid leaching (as evidenced by the detection of kaolinite, alunite, and ferrous clays) and the deposition of an anhydrous dark capping unit of volcanic/pyroclastic origin during the early Hesperian (3.7 Ga).

Possible formation mechanisms for the phyllosilicate-rich deposits are the alteration of volcanic ash layers, aeolian, or fluvial sediments in a wet environment, either because of top-down leaching in a pedogenesis context or through concurrent weathering and sedimentation (Gross *et al.*, 2016). Given that the dark capping layer is relatively resistant to erosion, it is expected that the main target outcrops will be well preserved.

## 8. Conclusions

In this work, we have tried to show that microorganisms could have appeared and flourished on early Mars, as they did on our planet. To maximize our chances of finding signs of past life, we must target the “sweet spot” in Mars' geological history, the one with highest lateral water connectivity, the early Noachian, and look for large areas preserving evidence of prolonged, low-energy, water-rich environments: the type of habitat that would have been able to receive, host, and propagate microbes. Finding signs of their possible existence would be a very important discovery, although ultimately we would want to understand to what extent their biochemical nature was similar to ours: Did Mars life have an independent genesis or do we share a common ancestor (McKay, 2010)?

1. The ExoMars rover's design, payload, and exploration strategy focus on the search for extinct life; however, the mission also has the potential to recognize chemical indicators of extant life. Only if we were to detect abundant, nondegraded, primary biomolecules—as one would expect to find in association with living (or recently deceased) microorganisms—could we postulate the possible presence of extant life in the samples we have analyzed. Considering the harsh near-surface conditions on Mars, and the fact that we are targeting low-latitude, relatively water-poor landing sites, we do not believe we have high chances of encountering active life. We mention this payload capability because the possibility, although small, exists.

The rover will be equipped with a drill to collect material from outcrops and at depth down to 2 m. This subsurface sampling capability is quite unique and provides the best chance yet to gain access to well-preserved chemical biosignatures.

2. Using the Pasteur instruments, the ExoMars science team will conduct a holistic search for biosignatures (morphological and chemical) and seek corroborating geological context information.

Although SAM's means to characterize indigenous organics have been hindered by their reaction with oxychlorine species in the martian soil, we have learned much from Curiosity to help us prepare future investigations. In fact, the ExoMars organics detection instrument, MOMA, is a joint undertaking of the SAM and COSAC (Rosetta lander) teams. Our work shows that the laser-desorption extraction method implemented in MOMA is not affected by perchlorates. In other words, we are able to detect (relatively large) organic molecules quite effectively even when oxychlorine species are present in the sample.

Another powerful capability of ExoMars is that it can investigate the same mineral grains with LDMS, VIS-IR, and Raman, allowing us the opportunity to observe a target with all three techniques, although the MOMA LDMS footprint is larger than that of the RLS and MicrOmega spectrometers.

Unfortunately, the Mars 2020 rover does not include an analytical chemistry laboratory. Instead, this mission's ability to infer the presence of organics will rely on the use of Raman spectrometry. The remote Raman on the mast is the perfect complement of laser-induced breakdown spectroscopy for the mineralogical–geochemical characterization of the martian surface, but it will be difficult to obtain organic molecule signals with this instrument. The Deep UV Raman technique on the robotic arm can help establish whether organics are present (*e.g.*, by identifying molecular functional groups and, in many cases, their fluorescence spectral features) (Beegle *et al.*, 2016). It will not be easy, however, to determine which are the exact molecules responsible for the observed signature and, hence, try to establish whether they are biogenic. Having a detailed organics characterization instrument (like SAM or MOMA) to complement the Raman spectrometers' findings would have greatly enhanced Mars 2020's ability to study possible biosignatures. Caching of samples was considered the higher priority for this mission.

3. Targeting an early Noachian location will grant us access to deposits of an age no longer available for study on our own planet. The absence of plate tectonics on Mars increases the probability that rapidly buried ancient sedimentary rocks (possibly hosting microorganisms) may have been spared thermal alteration and been shielded from ionizing radiation damage until denuded relatively recently. The scientific quality of the landing site in terms of suitable age; nature, duration, and connectivity of aqueous environments; sediment deposition, burial, diagenesis, and exhumation history will play a determinant role in shaping the mission's outcome.

4. We suggest categorizing the habitability of a candidate landing site in terms of the extent and frequency of liquid water lateral connectivity between the potential (micro)habitats.

5. We propose a possible scoring system for assigning a confidence value to a group of observations aiming to establish whether a location hosted life.

We find there is value in defining a set of measurements and rules to guide our preparations and help with the interpretation of any findings once on Mars. For example, when planning laboratory experiments to test our instruments, particularly MOMA, we can check to what extent our ability to verify the chemical biosignatures in the model is affected by (1) components mixed in with the sample; (2) type and age of rocks we analyze; (3) parameters (*e.g.*, temperature and mineral assemblage size) at the time of sample acquisition (drilling), processing (crushing), and distribution (dosing); and (4) decisions on measurement protocols (*e.g.*, the maximum temperature to use for the various types of MOMA ovens).

The model should be discussed, validated, and improved. It will be important to have such a tool in use before beginning the search for life on Mars. A definitive detection would entail the simultaneous verification of several biosignature attributes. We present four examples to show that this will not be easy.

6. We believe it is necessary to utilize terrestrial analogues to achieve a maximum *ExoMars Biosignature Score* as a “baseline” against which to compare mission results. We recommend that samples of the oldest Earth rocks known to have hosted life, for example, from the Pilbara and Barberton, be chemically analyzed with MOMA.

7. Life-seeking missions to other planets should target as many biosignatures indicated in Figure 3 as possible. We claim that their discoveries will not be conclusive unless such missions include powerful analytical chemistry capabilities that can allow for the unambiguous identification of key biosignatures of biomolecules and their degradation products.

The ExoMars rover is very well suited to search for signs of life. Nevertheless, the ultimate confirmation of a collection of potential biosignature detections may require more thorough analyses than can be performed with our present robotic means. Even a tentative finding would constitute a powerful catalyst for an MSR mission. Because of the ExoMars rover's special ability to explore the third dimension—depth—its discoveries will contribute immensely to determining what types of samples we should return to Earth.

## Abbreviations Used

ADRON: active detector for gamma rays and neutrons
ALD: analytical laboratory drawer
CDF: concurrent design facility, where new missions are studied in ESA
CLUPI: close-up imager, accommodated on the drill box's external surface
CS: crushing station
CSTM: core sample transport mechanism
Der: derivatization, an agent added to sample material that reacts with indigenous organic molecules to reduce their polarity, rendering the resulting compounds more volatile
DM: descent module: the capsule that enters the atmosphere for landing
DMF-DMA: *N*,*N*-dimethylformamide-dimethylacetal, one of the MOMA derivatization agents
EC: experiment cycle
EDM: entry, descent, and landing demonstrator module: part of ExoMars 2016
FOV: field of view
GCMS: gas chromatograph mass spectrometer: one of MOMA operation modes
GPR: ground-penetrating radar
HRC: high-resolution camera, part of PanCam
IR: infrared
ISEM: infrared spectrometer for ExoMars, on the rover mast
LDMS: laser desorption mass spectrometry: one of MOMA operation modes
LSSWG: Landing Site Selection Working Group
Ma_MISS: Mars multispectral imager for subsurface studies, the IR spectrometer integrated in the drill
MAVEN: Mars Atmosphere and Volatile Evolution Mission
MER: Mars Exploration Rovers, Spirit and Opportunity
MicrOmega: micro observatoire pour la mineralogie, l'eau, les glaces et l'activité
MOLA: Mars orbiter laser altimeter: an instrument on NASA's 1996 Mars Global Surveyor. 0-MOLA altitude is considered the reference elevation for Mars
MOMA: Mars organic molecule analyzer, in the rover's analytical laboratory
MSL: Mars Science Laboratory: NASA's Curiosity rover
MSR: Mars sample return
MTBSTFA/DMF: *N*-tert-butyldimethylsilyl-*N*-methyltrifluoroacetamide/dimethylformamide as a 3:1 mixture, one of the MOMA derivatization agents
PAHs: polycyclic aromatic hydrocarbons
PanCam: panoramic camera, on the rover mast
Pyr: pyrolysis, heating a sample to release volatile (fragments) organic molecules
RLS: Raman laser spectrometer, accommodated in the rover's analytical laboratory
ROCC: Rover Operations and Control Center
RSM: reference surface mission
SAM: sample analysis at Mars, the organics detection instrument on NASA's Curiosity rover
SP: surface platform: The element of the ExoMars 2018 DM reaching the surface. After rover egress, the SP becomes a science station
SPDS: sample preparation and distribution system
TGO: Trace Gas Orbiter: part of ExoMars 2016
TMAH: 25 wt% tetramethylammonium hydroxide in methanol, one of the MOMA derivatization agents
ToF-SIMS: time-of-flight secondary ion mass spectrometry
TV: thermal volatilization, sometimes also called pyrolysis, refers to the release of volatile organic molecules (fragments) by heating a sample
UCZ: ultra clean zone, the part of the ALD enclosing the sample path
UHF: ultra high frequency. The radiofrequency band used at present for rover-to-orbiter communications, sometimes called “proximity link”
VIS + IR: visible (red, green, and blue) plus infrared
VS: vertical survey
WAC: wide angle cameras in PanCam
WISDOM: water, ice, and subsurface deposit observations on Mars, a GPR
